# Addressing Key Questions in Organoid Models: Who, Where, How, and Why?

**DOI:** 10.3390/ijms242116014

**Published:** 2023-11-06

**Authors:** María Gómez-Álvarez, Marcos Agustina-Hernández, Emilio Francés-Herrero, Adolfo Rodríguez-Eguren, Clara Bueno-Fernandez, Irene Cervelló

**Affiliations:** 1 Instituto de Investigación Sanitaria La Fe (IIS La Fe), IVI Foundation, IVIRMA Global Research Alliance, 46026 Valencia, Spain; maria.gomez@ivirma.com (M.G.-Á.); marcos.agustina@ivirma.com (M.A.-H.); emilio.frances@ivirma.com (E.F.-H.); adolfo.rodriguez@ivirma.com (A.R.-E.); clara.bueno@ivirma.com (C.B.-F.); 2 Department of Pediatrics, Obstetrics and Gynecology, Universitat de València, 46010 Valencia, Spain

**Keywords:** 3D culture, organoids, hydrogel, bioengineering, female reproduction

## Abstract

Organoids are three-dimensional cellular structures designed to recreate the biological characteristics of the body’s native tissues and organs in vitro. There has been a recent surge in studies utilizing organoids due to their distinct advantages over traditional two-dimensional in vitro approaches. However, there is no consensus on how to define organoids. This literature review aims to clarify the concept of organoids and address the four fundamental questions pertaining to organoid models: (i) What constitutes organoids?—The cellular material. (ii) Where do organoids grow?—The extracellular scaffold. (iii) How are organoids maintained in vitro?—Via the culture media. (iv) Why are organoids suitable in vitro models?—They represent reproducible, stable, and scalable models for biological applications. Finally, this review provides an update on the organoid models employed within the female reproductive tract, underscoring their relevance in both basic biology and clinical applications.

## 1. Introduction—Organoid Definition

For decades, two-dimensional (2D) in vitro cell culture models were widely used for basic science and therapeutic applications, but the limitations of these techniques have become discernible. Specifically, conventional cell cultures are deprived of the native tissue-specific characteristics that are crucial for replicating in vivo cell behavior. This deficiency arises from the lack of the surrounding extracellular matrix (ECM), specific biochemical cues, and environmental stimuli [1]. In this regard, there is an unmet need to develop advanced in vitro cell culture platforms. The complexity of in vitro models has evolved, with three-dimensional (3D) cellular models preserving the biological features of the native organ or tissue. Indeed, organoid cultures are gaining popularity because they mimic the tissue’s physiology more accurately than other models [2]. By definition, organoids are organ-like structures that exhibit the native characteristics of their tissue of origin, including their morphological features, functional activities, and personalized treatment responses [3]. Remarkable advantages of organoids over traditional 2D cell cultures include the maintenance of cell–cell and cell–ECM interactions, the genotypic and phenotypic characteristics of the original tissue or organ, the heterogeneity of the original cells, and a significantly extended lifespan [4].

The term organoid has been indiscriminately employed by the scientific community in recent years. Based on its etymological roots (the Greek suffix *-oid* denotes *similar to*), organoids could be defined as cellular structures that resemble native tissues and organs but grow under specific in vitro conditions [5]. Although there is no consensus, most authors define organoids as 3D cellular structures derived from a multitude of a single type of organ-specific cells, embedded in an extracellular scaffold [6]. Within this microenvironment, the cells self-organize into 3D constructs that mimic the native tissue structure, function, and characteristics. Notably, organoids can be subclassified according to their morphology: spherical-shaped organoids (which, as argued herein, are inaccurately referred to as spheroids); tube-shaped organoids (tuboids) [7]; somite-shaped organoids (somitoids) [8]; and star-shaped organoids [5]. Another traditional classification is based on the cellular material of organoids, which are typically divided into organoids derived from induced pluripotent stem cells (iPSCs) or embryonic stem cells (ESCs) and organoids derived from adult stem cells (ASCs) [2]. However, we have updated this classification, given the recent description of organoids derived from differentiated somatic cells [9] and stable cell lines [10], as will be discussed in Section 2.

**Figure 1:**
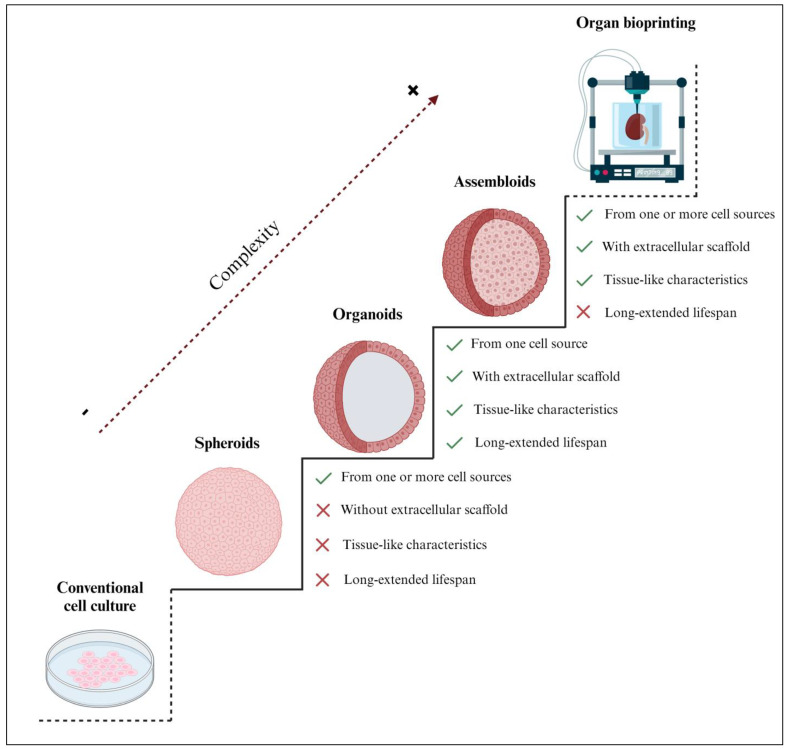
Comparison of the structural complexity of 3D spheroids, organoids, and assembloids with respect to conventional 2D cell cultures and organ bioprinting. Organoids are considered the most suitable in vitro tissue engineering strategy, with only one cell type supported by an extracellular scaffold, mimicking the characteristics of the native tissue. Unlike spheroids and assembloids, organoids have a significantly extended lifespan; therefore, they offer the possibility of biobanking. Notably, although iPSC/ESC–derived organoids are generated from a single cell source (iPSCs or ESCs, respectively), they can give rise to various cell types, including epithelium, fibroblasts, and vasculature. Thus, their expansion capacity may differ from other organoid types but remains significantly higher than other 3D models. Created with BioRender.com (accessed on 2 November 2023).

Three-dimensional engineered tissue models increase in biological complexity from spheroids, organoids, and assembloids to entire bioprinted organs (Figure 1). As there is currently no established classification for spheroids, in this review, we define them as 3D spherical cell aggregates containing one or more cell types that were cultured without an extracellular scaffold [11]. Most of the organoids described in the current literature were defined as spheroids since they shared several biological properties; however, by our definition, not all spheroids are organoids, as some lack scaffolding and the ability to expand in vitro, or they may consist of multiple cell types (Figure 1). Notably, the main difference between spheroids and organoids is the capability of organoids to mimic the in vivo characteristics and behavior of the native tissue, while some spheroids are simpler; most spheroids cannot mimic effectively and have a shorter lifespan. Indeed, spheroids are often used for short-term experiments and are not cultured for extended periods [12,13], as is often the case with organoids [14,15,16]. Hence, 3D organoids offer distinguished in vitro models with a confirmed significantly extended lifespan and, therefore, the possibility of biobanking [17].

Alternatively, assembloids are defined as 3D cell aggregates, made from two or more cell types within an extracellular scaffold [18], and are often used to model tissue-specific cell interactions [19]. Some authors have erroneously described assembloids as spheroids; however, assembloid models are sophisticated multilayer or multi-tissue organoids. In some cases, the scientific literature has identified assembloids as spheroids because of their spherical shape. However, as far as we know, assembloids are more complex and are closer to in vivo organs [20]. Assembloid culture is presented as an underexplored yet promising approach in tissue engineering due to several technical issues that remain to be addressed. The current limitations not only include the different growth rates of each cell type but also the challenging integration of these cells into the extracellular scaffold [21]. Additionally, the inherent complexity of the in vitro propagation of assembloid models hinders their ability to expand, be cultured, and be biobanked [19].

The present review comprehensively addresses the four main considerations for organoid cultures: (i) What constitutes organoids?—The cellular material, which defines the type of organoid. (ii) Where do organoids grow?—The extracellular scaffold, which is essential for 3D conformation. (iii) How are organoids maintained in vitro?—Via the culture medium, which is essential for their adequate development and maintenance. (iv) Why are organoids suitable in vitro models?—Representing reproducible, stable, and scalable models for research applications, organoid cultures lend biological significance to these cellular models. Finally, we describe the advantages of developing and establishing human-derived organoid models for studies in reproductive biology and reproductive medicine.

Thus, their expansion capacity may differ from that of other organoid types but remains superior to that of other three-dimensional cell models.

## 2. What Constitutes Organoids? The Cellular Material

One of the key components of organoid culture is the cellular material, which can be obtained from different sources. Organoids can be derived from most tissues and organs of the human body and, thus, have the potential to model an ample array of health and disease conditions [2]. However, the biological characteristics of these conditions and the stages of in vitro culture depend on the cellular composition. For example, hepatic and endometrial organoids are cultured for approximately 7–10 days [22,23], whereas brain [24] and intestinal [25] organoids can be maintained in vitro for up to 15–20 days.

Given the vast range of the reported cellular constituents of organoids, we categorized the cells based on their origin and the methodologies employed to generate organoids. This classification includes pluripotent stem cells, primary cells, and cell lines.

### 2.1. Pluripotent Stem Cells

**Figure 2:**
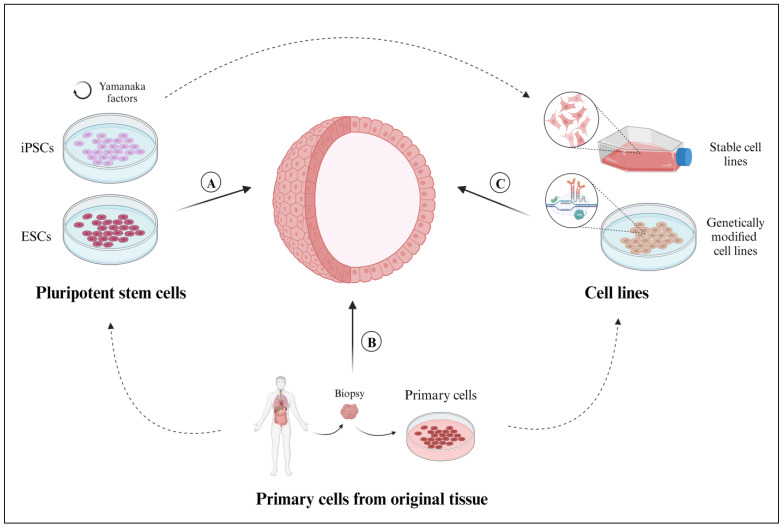
Sources of cells employed for the development of 3D organoid models. (**A**) Primary cells (tissue-resident ASCs, progenitor cells, or differentiated somatic cells), isolated from healthy or diseased tissue samples. (**B**) Pluripotent stem cells derived from embryonic stem cells (ESCs) or pluripotent reprograming of somatic cells (induced pluripotent stem cells (iPSCs)). (**C**) Cell lines, whether established in the laboratory or available through commercial vendors or biobanks, can be genetically modified by using CRISPR-Cas9 technology. Created with BioRender.com (accessed on 2 November 2023).

Pluripotent stem cells (PSCs) are capable of self-renewing and differentiating into any type of cell in the human body [26] (Figure 2a). Using PSCs in organoid culture confers the notable benefit of introducing culture supplements to the direct differentiation of diverse cell lineages. For example, PSCs supplemented with FGF4 differentiated into intestinal cells and gave rise to intestinal organoids [27]. However, PSCs are unable to mimic the native tissue characteristics as effectively as primary cells.

The two main sources of PSCs are ESCs, which are derived from the inner cell mass of blastocysts [28], and iPSCs, which are obtained from a formerly differentiated somatic cell and then reprogrammed into pluripotent stem cells using Yamanaka factors (Oct3/4, Sox2, Klf4, and c-Myc) [29]. ESCs originate from a commercial cell line (e.g., H9 [24,30] and WA09 [31]), whereas iPSCs can be obtained from both commercial cell lines [32,33,34] and primary tissue samples [35,36,37].

The scientific literature describes two different approaches for generating organoids with PSCs. The first method consists of maintaining PSCs in monolayer cultures until a 70–90% confluence is reached [38]. Under suitable culture conditions, these PSCs self-organize into 3D aggregates, generally referred to as embryo bodies (EBs) [39], which are transferred into an extracellular scaffold to form these organoids. The other method consists of generating them from individual PSCs grown in conventional 2D cultures. In this second approach, EB formation is prevented by adding rho-associated kinase pathway inhibitors (ROCK), such as Y-27632, to the culture medium prior to embedding the individual PSCs in an extracellular scaffold to generate the organoids [40].

### 2.2. Primary Cells Isolated from Original Tissues

Organoids that are derived from primary cells isolated from fetal or adult tissue will faithfully preserve inherent biological features, including the secretion of paracrine factors and cell-cell communications (Figure 2b). However, the main disadvantages of deriving organoids from primary tissues include the invasiveness of tissue biopsies and the technical complexity of cell isolation, as described below.

To process tissue samples for cell isolation, the tissue layers are mechanically separated and the tissue of interest is cut into smaller pieces (pieces of 1 mm [41] and 2 mm [42] in length) to facilitate the enzymatic digestion of the ECM. Despite the variable efficacy of the dissociation processes across tissue types, it is possible to add enzymatic cocktails containing collagenase to degrade the collagen fibers in bladder cancer [43], trypsin to catalyze the hydrolysis of peptide bonds in adenomas [44] and peripheral lung tissues [45], and/or dispase to hydrolyze fibronectin and collagen IV in hair follicle [42] and colorectal cancer [46] organoids. Both the digestion time and temperature differ according to the characteristics of the primary tissue. In some cases, DNase is added to degrade the residual DNA released from necrotic cells. Next, tissue-resident ASCs, progenitor cells, or differentiated somatic cells [9] from healthy or diseased tissues can be isolated for organoid formation [2] via fluorescence- or magnetic-activated cell sorting, based on biomarker expression, cell shape, or size [47,48], or via filtering through 40-μm [45], 70-μm [49], or 100-μm [45] cell strainers. The isolated cells are cultured in vitro within an extracellular scaffold that supports their self-organization and development. As will be reviewed herein, each type of organoid requires a particular cell/scaffold ratio and an adequate culture medium.

### 2.3. Cell Lines

Organoids can also be derived from commercially available, previously established, or biobanked cell lines (Figure 2c). The cells are cultured in a monolayer with their corresponding culture media until a certain confluency (80%) is reached, then they are collected and cultured with an extracellular scaffold to promote organoid formation [37]. Given their extended lifespan, the organoids can usually be preserved in multiple passages. Cell lines capable of forming organoids include the BT-474 commercial cell line for breast cancer organoids [46], the LIM1863 commercial cell line for colon cancer organoids [10], and the BTS5 and BTS11 trophoblast stem cell lines for trophoblast organoids [50]. While using stable cell lines certainly expedites organoid generation, these immortalized cells might not respond as realistically as primary cells collected directly from individual patients.

In addition, organoids can also be made from cells that have been genetically modified and established as a stable, genetically modified cell line (Figure 2c). Genetically modified cell lines are frequently used to study human diseases because they mimic the responses and characteristics of mutated cells. Within the context of organoid applications, the overexpression or depletion of specific proteins facilitates the study of functional changes within the native tissue. Genetically modified organoids not only provide a valuable platform for personalized drug testing but can also be used to study rare genetic diseases [51]. To generate genetically modified organoids, single guide RNAs (gRNAs) are designed to target specific genes using CRISPR-Cas9 technology [52]. The gRNAs are introduced into the cells via electroporation [53] or transfection [54], either during the initial 2D culture [51] or after the organoids are generated and cultured in their corresponding 3D scaffold [52,53]. However, like any cell lines undergoing genetic modification, there is a substantial loss of cellular material; maintaining a long-term culture remains a notable drawback, resulting in a significantly low efficiency for this technique [55].

As described above, the source and processing of cellular material are essential for the establishment, maintenance, and expansion of organoids. However, in all cases, the 3D constructs require structural support from extracellular components and an appropriate culture medium to nourish cells. For this reason, in the following sections, we will look deeper into the Where? and How? questions about organoid culture approaches.

## 3. Where Do Organoids Grow? The Extracellular Scaffold

**Figure 3:**
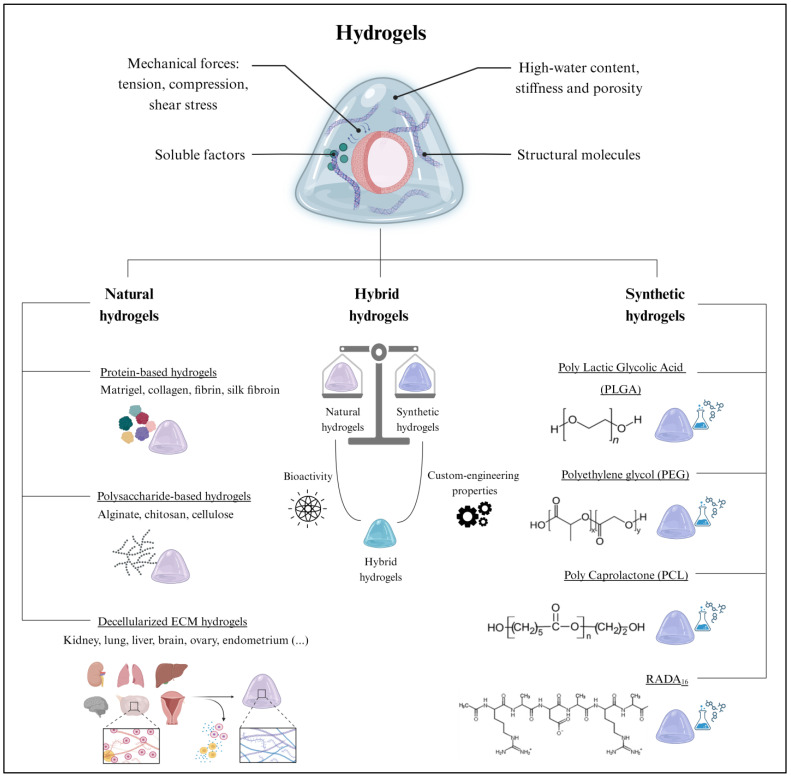
The extracellular scaffold in organoid culture. (**Top**) Hydrogels are extensively employed for organoid culture due to their remarkable biological properties. (**Bottom**) Hydrogels can be classified as natural, hybrid, or synthetic, which all differ in composition and their inherent properties. Created with BioRender.com (accessed on 2 November 2023).

Extracellular scaffolds provide support for organoid growth, development, and maintenance through mechanical and biochemical cues [56]. The tension, compression, and shear stress provided by the ECM influence cell proliferation, differentiation, and migration, while structural proteins maintain cell–cell contact and soluble factors promote cell–ECM interactions, which are crucial for maintaining cell architecture and function [57] (Figure 3).

Hydrogels are the leading type of extracellular scaffold used for 3D organoid culture. Hydrogels are cross-linked hydrophilic polymer networks with high water content and physicochemical properties closely resembling those of native tissues [58]. Remarkably, the potential to adapt certain hydrogel properties, such as stiffness and porosity, allows hydrogels to be tailored to specific organoid requirements [59]. Hydrogels also protect organoids from external stresses, while simultaneously allowing the exchange of nutrients and oxygen [60]. Emerging alternatives to hydrogels include microspheres [61] and porous scaffolds [62], among other approaches [63].

However, the present study focuses on 3D support based on hydrogels, due to their established and worldwide use. While numerous classifications for hydrogels have been proposed in the literature [58], here, we broadly categorize them based on their natural or synthetic origin. However, within each of these groups, hydrogels can be further subclassified according to their molecular composition.

### 3.1. Natural Hydrogels

Historically, natural hydrogels were extensively applied in tissue engineering due to their biocompatibility, biodegradability, and low toxicity [58]. Their macromolecules provide functional support for cell growth and proliferation [64]. Among the different biomaterials, proteins and polysaccharides are suitable for generating natural hydrogels. Furthermore, hydrogels can be formed by a complex mixture of both components, as in the case of decellularized ECM hydrogels (Figure 3).

#### 3.1.1. Protein-Based Hydrogels

Protein-based hydrogels are the predominant class of biomaterials employed in tissue engineering [65]. These hydrogels are primarily composed of collagen, fibrin, and silk fibroin, among other natural proteins [65], which possess unique biochemical properties that contribute to the functionality of the hydrogel. Sometimes, a mix of structural proteins is used, such as Matrigel, which is considered the gold standard biomaterial for organoid culture. Matrigel is derived from the basement membrane matrix of Engelbreth–Holm–Swarm (EHS) mouse sarcomas. It contains a complex blend of ECM proteins, growth factors, and other bioactive molecules that provide a supportive microenvironment for organoid development [66]. Matrigel has been extensively utilized to culture intestinal [67], hepatic [68], pancreatic [69], ovarian [70], prostatic [71,72], and endometrial organoids [22,73]. Similarly, collagen-based hydrogels, derived from the most abundant proteins in the ECM, provide a natural environment for organoids to grow and differentiate. Several studies have demonstrated the ability of collagen-based hydrogels to support the generation of intestinal [74], liver [75], lung [76], and brain organoids [77], which successfully replicate the architecture and functionality of their corresponding in vivo tissues. Moreover, fibrin hydrogels have also been applied to bioengineer kidney organoids, promoting endothelial cell infiltration and capillary formation within the hydrogel [78]. Fibrin hydrogels are formed by the thrombin-mediated enzymatic crosslinking of fibrinogen into fibrin. Thus, the gel formation process can be controlled to modulate the gelation kinetics [79]. Finally, silk hydrogels, derived from silk fibroin, were also suitable for the growth of brain [80] and intestinal organoids [81]. Silk hydrogels can be prepared using different techniques, such as self-assembly or crosslinking methods, to attain the desired gel structure and properties [82].

#### 3.1.2. Polysaccharide-Based Hydrogels

Polysaccharide-based hydrogels are mainly based on alginate [83] and chitosan [84], but cellulose hydrogels have also been described with less frequency [85]. Alginate, which is characterized by the presence of guluronic and mannuronic acid residues, exerts precise control over the release kinetics of encapsulated growth factors and nutrients, thereby enhancing the viability and functionality of organoids [86]. For example, alginate hydrogels promote the 3D organization of neural cells into organoids and support the assembly of vascular-like systems in co-cultures with endothelial cells [87]. Conversely, chitosan is a cationic polysaccharide that improves the encapsulation efficiency of negatively charged molecules [88]. This cationic nature contributes to the sustained release of biomolecules, which is particularly relevant for in vitro organoid cultures. Indeed, chitosan hydrogels were recently employed as a 3D platform for different organoid models, including gut organoids [84,89].

#### 3.1.3. Decellularized Extracellular Matrix Hydrogels

Decellularized ECM hydrogels are obtained from the decellularization of organs or tissues, a technique that removes cellular components while preserving the structural and functional proteins of the ECM (glycosaminoglycans, proteoglycans, and growth and soluble factors) [90]. These natural ECM hydrogels have been derived from kidney [91], lung [92], liver [93], brain [94], ovary [95], and endometrial tissue [23], among others. Once decellularized, the tissues are milled, lyophilized, solubilized, and neutralized to form a pre-gel solution [90]. Subsequent incubation at the physiological temperature (37 °C) or the addition of acetic acid to decrease the pH to 4.0 [96] induces the spontaneous 3D remodeling of the monomeric components’ intramolecular bonds (e.g., collagen reorganizes into complex fibers). The polymerization kinetics are influenced by the native biochemical profile of the source tissue, as well as the proteins remaining after decellularization [96]. The specific biomolecules secreted by each tissue’s resident cells determine the organization and biochemical composition of the corresponding decellularized ECM hydrogels [97]. Unlike Matrigel, decellularized ECM hydrogels retain the full biochemical complexity of the native tissue; thus, they are presented as promising biomaterials, not only for culturing organoid models in vitro but also for mimicking the native tissue environment [90].

### 3.2. Synthetic Hydrogels

Synthetic hydrogels emerged as valuable tools in organoid culture due to their fully customizable mechanical and biochemical properties and their versatility (Figure 3) [98]; they encompass a broad range of biomaterials with varying compositions, crosslinking mechanisms, degradation rates, and rheological properties [98]. The classification parameters of synthetic hydrogels include the polymer type, the crosslinking method, swelling behavior, and biological interactions. Some recently reported synthetic hydrogels were based on polylactic glycolic acid (PLGA), polyethylene glycol (PEG), polycaprolactone (PCL), and RADA_16_ (commercialized as PuraMatrix) (Figure 3). In particular, PLGA hydrogels, characterized by their excellent biocompatibility and biodegradability, were employed for intestinal and liver organoid cultures [99,100,101]. Similarly, the slow degradation of PCL hydrogels was useful for the prolonged culture of neural organoids [102]. In contrast, PEG hydrogels, known for their high water content and modifiable crosslinking, facilitated nutrient and oxygen diffusion to intestinal organoids [103]. Finally, RADA_16_-based hydrogels favored the formation of complex 3D neural structures in brain organoids [104,105].

Notably, natural and synthetic biomaterials can be combined to create hybrid hydrogels that capitalize on the advantages offered by different biomaterials (Figure 3) [106]. Indeed, hybrid hydrogels strike a balance between the bioactivity of natural hydrogels and the custom-engineering properties of synthetic hydrogels, resulting in improved organoid growth, stability, viability, maintenance, and functionality [106].

## 4. How Are Organoids Maintained In Vitro?—The Culture Medium

**Figure 4:**
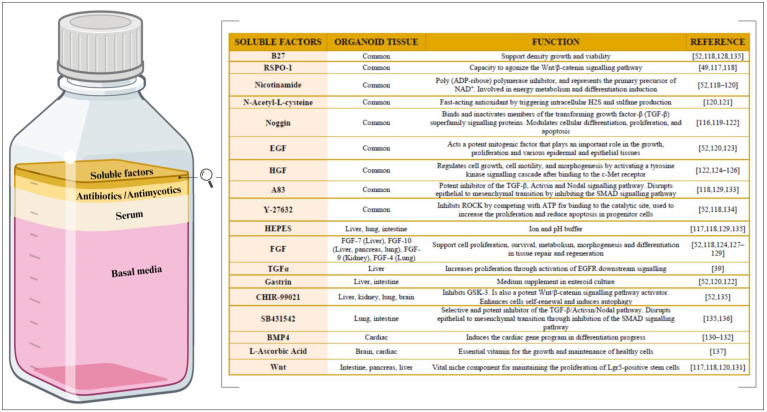
Composition of culture medium for organoid culture. (**Left**) The relative proportions of the basal media, serum, antibiotics/antimycotics, and soluble factors within the organoid culture medium. Created with BioRender.com. (**Right**) The main soluble factors, along with their corresponding biological function(s). R-Spondin-1 (RSPO-1), epithelial growth factor (EGF), hepatocyte growth factor (HGF), 3-(6-Methyl-2-pyridinyl)-N-phenyl-4-(4-quinolinyl)-1H-pyrazole-1-carbothioamide (A83), 2-[4-(2-hydroxyethyl)piperazin-1-yl]ethane-1-sulfonic acid (HEPES), fibroblast growth factor (FGF), transforming growth factor alpha (TGFα), and bone morphogenetic protein 4 (BMP4).

Besides an extracellular scaffold, in vitro organoid culture requires a rich source of essential nutrients, growth factors, and signaling molecules—vital for the sustained growth, maintenance, and proliferation of the organoid—which standard culture media does not provide (Figure 4). However, it is important to note that culture media are not universally standardized in the organoid field; instead, they are intricately customized to meet unique tissue-specific organoid demands. Establishing an appropriate culture medium is a critical prerequisite for successful organoid culture, as it directly influences the fidelity of organoid establishment, development, maintenance, functionality, and responses.

Different cell types and stages of development have distinct culture requirements. Thus, defining the culture medium that meets their specific needs can optimize the organoids’ formation, survival, metabolic functions, and differentiation [107]. In this regard, the organoid culture medium has four main components: the basal media, serum, antibacterial and antimycotic agents, and soluble factors (Figure 4).

### 4.1. Basal Medium

Generally, most of the volume of the culture medium is made up of basal medium (Figure 4). The main function of the basal medium is to provide the cells with a suitable environment for growth, promoting the survival, preservation, and development of cultured cells [108]. In addition, it maintains the homeostasis of the culture by acting as a buffer for any changes that may affect the cells, such as a change in the pH of the medium. To this purpose, all basal media share a common cocktail of essential components, such as numerous inorganic salts, sugar, essential amino acids, and water-soluble vitamins [109]. Moreover, some media contain additional factors to support the biological requirements of certain cell cultures. For example, commercial media like α-MEM, DMEM, and Ham’s F-12 are suitable for adherent cell cultures, whereas the RPMI 1640 culture medium is recommended for suspended cell cultures [109].

### 4.2. Serum

Additionally, an important part of the medium composition is the serum (Figure 4). The main function of serum is to provide a source of nourishment for the cells, including the active biomolecules necessary for cell survival and growth, such as amino acids, proteins, vitamins, carbohydrates, lipids, hormones, growth factors, inorganic salts, and trace elements [109]. Serum also enhances the pH-buffering capacity of the culture medium and reduces physical damage to the cells when used as a basal medium [110]. While fetal bovine serum (FBS) remains the most popular serum employed, the use of fetal calf and horse serum has also been reported [111,112].

### 4.3. Antibiotics and Antimycotics

Bacterial or mycotic contamination are major concerns in cell culture (Figure 4). Contamination may arise from various sources, including the researcher and/or the laboratory environment, other cells in the laboratory, and reagents [113]. To avoid contamination, antibacterial and antimycotic compounds are typically incorporated into the culture media. The most frequently used antibiotics are penicillin and streptomycin. Penicillin represents a class of antibiotics that eradicates Gram-positive bacteria by inhibiting peptidoglycan synthesis in the bacterial cell wall [114]. Conversely, streptomycin inhibits bacterial protein synthesis by binding to the small 16S rRNA of the 30S subunit of bacterial ribosomes, interfering with codon reading and ultimately inducing cell death [114]. Regarding the popular antimycotics, amphotericin binds to the ergosterol present in the fungal cell membrane, leading to the formation of pores that compromise cell membrane integrity, facilitate ion loss, and, ultimately, induce fungal cell death [115].

### 4.4. Soluble Factors

Soluble factors are arguably the most important component of organoid culture medium. Soluble molecules are capable of binding to the cellular receptors, triggering permissive or inhibitory intracellular signals to initiate cell differentiation and/or proliferation (Figure 4). The soluble molecules supplemented in most in vitro cultures predominantly consist of the following growth factors and proteins [116]: R-Spondin-1 (RSPO-1) [49,117,118], nicotinamide [52,118,119,120], N-acetyl-L-cisteyne [120,121], noggin [116,119,120,121,122], epithelial growth factor (EGF) [52,120,123], hepatocyte growth factor (HGF) [122,124,125,126], fibroblast growth factor (FGF) [52,118,124,127,128,129], transforming growth factor alpha (TGFα) [39], gastrin [52,120,122], bone morphogenetic protein 4 (BMP4) [130,131,132], and Wnt [117,118,120,131]. However, some small-molecule drugs such as A83 [118,129,133], Y-27632 [52,118,134], CHIR-99021 [52,135], SB431542 [135,136], and L-ascorbic acid [137] have also been reported. Complementary buffers that maintain the pH level and enhance cell viability and function include the following: B27 [52,118,128,135], HEPES (2-[4-(2-Hydroxyethyl) piperazin-1-yl]ethane-1-sulfonic acid) [117,118,129,135]. Growth factors may be costly and unstable, while small-molecule drugs can affect the off-target pathways, resulting in poor reproducibility. Thus, one study experimented with combining biologics and small-molecule drugs in organoid cultures and obtained positive results [138]. Despite the considerable spectrum of variability and the specificity of soluble factors in culture media, Figure 4 highlights how different types of organoids have benefited from certain factors. Once the three main components (cellular material, ECM scaffolding, and the specific medium) of organoid culture have been explored in depth in this review, some of the applications of these 3D cell models will be examined. As already discussed, organoid technology has emerged as a revolutionary tool in biological research, offering a unique and dynamic platform to study in detail the many complex cellular interactions and tissue-specific functions in vitro.

## 5. Why Are Organoids Suitable In Vitro Models?—General Biomedical Applications

Organoid-based bioengineering strategies have led to significant advancements in tissue biobanking [101], modeling the in vivo characteristics of human pathologies [6,139], creating novel platforms for drug discovery and screening [140], and improving the efficacy of personalized medicine [141].

### 5.1. Biobanking

A biobank is defined as a systematic collection of human biological samples and the associated data, intended for research purposes [142]. This concept was driven by the pressing need for sustainable and reproducible sources of human tissue samples, which currently have limited availability, are often collected invasively, and are subject to considerable inter-individual variability [139]. Organoids are considered an ideal candidate for biobanking due to their self-renewing capacity, significantly extended lifespan, and ability to maintain tissue-specific features over extended periods of time [1]. Biobanked, patient-derived organoids can be employed to identify individualized therapeutic strategies, as well as predict the response to drug treatments based on a patient’s genetic and phenotypic profiles [143]. Furthermore, these repositories give researchers worldwide access to a broad range of standardized and quality-controlled samples, promoting collaborative research and minimizing inter-study variations. The Human Cancer Models Initiative (HCMI) is a notable example of a global effort to establish a comprehensive biobank of cancer organoid models (i.e., colon, brain, and breast cancer organoids), making these valuable resources widely available to the scientific community [144]. Nevertheless, the limitations of biobanking include the inherent high cost and the technical complexity related to establishing these repositories [143]. Despite these challenges, the continuous refinement of biobanking techniques and collaborative efforts in establishing large-scale biobanks hold the potential to revolutionize biological research and advance our understanding of human health and disease.

### 5.2. Disease Modeling

Organoids represent a powerful and versatile platform from which to study myriad pathological conditions (e.g., infectious or genetic diseases and cancer). This approach originated from the need to bridge the gap between in vitro and in vivo models for biomedical research. Many pathologies have a multifactorial origin, which cannot be modeled by static 2D in vitro culture conditions that do not reflect the regulatory factors produced by interactions between different cell types or peripheral tissues [140].

#### 5.2.1. Infectious Diseases

Organoids derived from different tissues are employed to investigate a wide range of infectious agents, including viruses and bacteria. The ability to model infectious diseases in organoid cultures has not only provided insights into host–pathogen interactions but has also helped improve the specificity of antibiotic and antiviral agents. For instance, respiratory organoids have been employed to model infections caused by respiratory viruses, such as influenza and respiratory syncytial virus [145]. Similarly, human intestinal organoids have been applied to study norovirus infection, providing valuable insights into viral replication and putative antiviral targets [146].

#### 5.2.2. Genetic Diseases

Targeted gene-editing technologies, like CRISPR/Cas9, have facilitated the modeling of genetic diseases in organoid culture. Several groups have successfully created organoid models with disease-specific mutations [17]. In particular, Dekkers et al. introduced a cystic fibrosis (CF) driver mutation into iPSCs using CRISPR/Cas9. The iPSCs were then differentiated into lung or intestinal organoids exhibiting the characteristics of CF-affected tissues, providing a platform by which to study CF pathology and test potential therapeutic approaches [147].

#### 5.2.3. Cancer

Various cancers are known to present intra-tumoral heterogeneity, with distinct cell populations harboring different genetic alterations and drug sensitivities [17]. Organoids derived directly from patient tumor samples successfully preserve the genetic and phenotypic heterogeneity observed in the original tumors [148], allowing researchers to study specific tumor subpopulations and identify potential targets related to tumor initiation and progression [149]. Notably, pancreatic [149], colorectal [148], and brain [150] cancer organoids represent a small subset of the numerous cancer organoids documented to date.

### 5.3. Drug Discovery and Toxicology Studies

The drug discovery process involves identifying and developing new treatments by exploring potential drug compounds, rigorously testing drug effectiveness and safety, and optimizing them for clinical use [151]. However, this process is time-consuming and places a substantial burden on the healthcare system. Human organoid models are significantly superior to conventional 2D cell cultures for use in drug discovery and toxicity screening since they provide physiologically accurate responses to treatments and expedite non-invasive patient-specific drug testing. Furthermore, organoids reduce the need for animal experimentation, providing a more cost-effective approach to drug testing while promoting ethical and sustainable research practices [152]. Drug discovery in organoid cultures is further distinguished by its efficiency, as organoids can be expanded and maintained in high-throughput formats, facilitating large-scale drug screening and/or the simultaneous testing of multiple therapeutic compounds.

### 5.4. Personalized Medicine

Personalized medicine is shifting healthcare paradigms by leveraging individual patient data to tailor preventive, diagnostic, and therapeutic management strategies [152]. Translating organoid models into clinical use will play a pivotal role in achieving these objectives, as patient-derived organoids can predict individualized responses to specific medications, allowing for more precise and targeted therapeutic strategies [153].

#### 5.4.1. Transplantation Therapy

Historically, transplantation therapy involved transferring viable cells, organs, or tissues to replace or restore the function of damaged tissues or organs. However, the development of donor-derived organoid models has had a remarkable positive impact on regenerative medicine. For instance, sweat gland organoids, transplanted into mice with dorsal injuries, contributed to the regeneration of their epidermis and sweat glands [154]. Nevertheless, for organoid transplantation to be considered viable for clinical application, their inability to form complex vascularized structures needs to be overcome [155].

#### 5.4.2. Immunotherapy

Understanding the interactions between the immune system and the tumor microenvironment is essential for designing targeted cancer approaches. As previously described, organoids mimic tumor heterogeneity in vivo and preserve the tumor’s microenvironment components, ensuring the interaction and dynamic connection between the tumor cells and their external environment. By incorporating immune cells into organoid systems, researchers can describe the effect of the immune cells on tumor organoids. For example, co-culturing organoids with effector T cells can result in an effective anti-tumor organoid immune response by decreasing the number of live organoid cells and increasing immune cell function [156]. As a result, these complex models promote the discovery of novel immunotherapeutic targets and/or the improvement of personalized treatments. Thus, organoid models also provide a more accurate bioplatform to test novel immunotherapeutic agents and optimize their use for individual patients.

#### 5.4.3. Gene Repairing

Gene repairing is based on gene-editing technologies and primarily focuses on rectifying the driver mutations of genetic diseases [17]. Employing organoid cultures for gene repair represents a transformative approach to tackling genetic diseases at the cellular level, enabling precise and individualized therapies for patients affected by genetic disorders. As an example, retinal organoids were employed to repair genetic mutations associated with inherited retinal degenerative disorders, providing a prospective avenue for future gene therapies aimed at treating vision loss [157].

## 6. Applications of Organoids Modeling the Female Reproductive Tract

**Figure 5:**
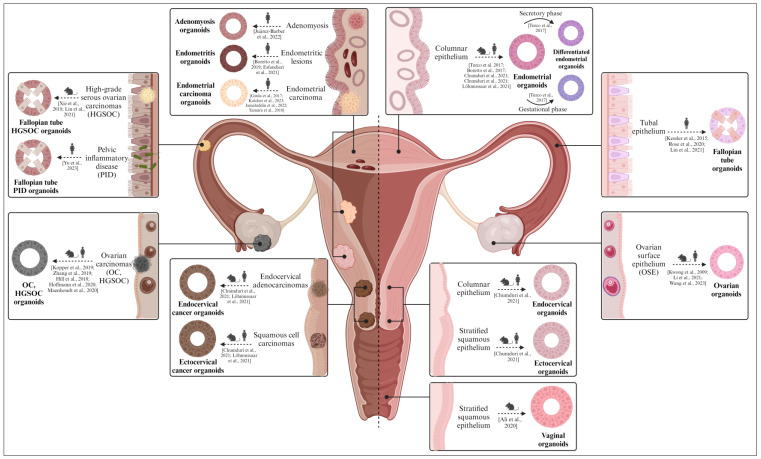
Diverse organoid models found in the female reproductive tract (from mice/rats and humans). Both healthy (**right**) and pathological (**left**) organoids derived from the ovary, fallopian tubes, endometrium, cervix, and vagina are described. Healthy organoids recapitulate the biological features of the native epithelium in vitro, while pathological organoids mimic the molecular and histological characteristics of the corresponding gynecological disease. Created with BioRender.com (accessed on 2 November 2023).

This section not only underscores the pivotal role of organoids in advancing the understanding of female reproductive health but also highlights their future applications in reproductive medicine and research. We discuss how organoids are catalyzing the understanding of reproductive disorders, expediting the screening of therapeutic interventions, and overall, substantially contributing to personalized medicine within the female reproductive system (Figure 5).

### 6.1. Ovary

The ovaries, commonly referred to as the female gonads, are responsible for housing oocytes and producing female sex hormones, like estrogen, progesterone, and inhibin, among others. The ovary comprises various types of cells, including superficial epithelial cells that form the human surface epithelium (OSE), germ cells (oocytes), granulosa cells, steroidogenic cells (granulosa and thecal cells), and ovarian stromal cells [158].

Kwong et al. were the first to develop organoid models derived from normal human OSE cells. In their study, primary cells sourced from healthy ovaries, which were suspended in a medium supplemented with 10% serum in Matrigel-coated wells, formed spheroidal structures that exhibited positive immunoreactivity for OSE markers (i.e., calreticulin and cytokeratins) [159]. However, these structures did not persist as long-term cultures. Conversely, ovarian organoids derived from female germline stem cells exhibited endocrine functions and produced oocytes in vitro, with low maturation rates [160,161].

In terms of pathological conditions, ovarian carcinoma is a complex ailment encompassing diverse tumor subtypes, each distinguished by specific genetic and pathological attributes. Kopper et al. derived organoids from OSE obtained from individuals who were highly susceptible to ovarian cancer (OC) due to germline mutations in breast cancer genes 1/2 (BRCA1/2) [14]. The OSE organoid lines were established with an efficiency surpassing 90% and demonstrated keratin 8 (KRT8)-positive expression, featuring the characteristic folds and invaginations. However, further research is imperative to enhance their capability for sustained propagation. In contrast, organoids originating from murine OSE have displayed the potential for extended passaging under specific culture conditions, containing a component of the Wnt/β-catenin signaling pathway (Wnt3a), R-spondin-1, noggin, EGF, nicotinamide, hydrocortisone, and β-estradiol [162]. Remarkably, organoids were also generated from high-grade serous ovarian carcinoma (HGSOC), the most prevalent and severe manifestation of ovarian cancer [163]. These organoids faithfully displayed the prominent morphological characteristics associated with HGSOC, including nuclear pleomorphism and a disordered epithelium; however, they had limited expansion capacity. A more recent investigation documented noteworthy enhancements in the HGSOC organoid system, culminating in the establishment of 15 distinct organoid lines that closely match the mutational profile and phenotype of the parental tumor [164]. The broad spectrum of OC subtypes was effectively recreated using organoid derivation, through the supplementation of compounds such as hydrocortisone, forskolin, and neuregulin 1 (NRG1) [14,165]. Overall, Kopper et al. remarkably succeeded in generating a substantial collection of fifty-six organoid lines, with 65% efficiency and 85% post-cryopreservation viability (a prerequisite for establishing a viable biobank) [14].

To this day, ovarian organoids have proven to be adaptable platforms for a range of scientific manipulations, including gene editing and drug sensitivity profiling [166,167], as evidenced by many studies [14,70,163,164,165,168,169,170]. The contemplation of more intricate organoid models (i.e., assembloids), with the potential integration of immune, stromal, and/or vascular components, kindles future endeavors to evaluate clinically pertinent pharmaceuticals that are aimed at targeting neo-angiogenesis and tumor immunology. This notion is underscored by findings demonstrating that short-term 3D organoid co-cultures of HGSOC and immune cells responded to immune checkpoint inhibitors [171].

### 6.2. Fallopian Tubes

The Fallopian tubes (FT), also referred to as the oviducts, connect the ovaries and the uterus, facilitate fertilization, and support the initial stages of embryo development. The FT consist of a thin layer of smooth muscle cells surrounding the stroma and the tubal epithelium, lined with secretory and ciliated cells [172].

In 2015, Kessler et al. pioneered healthy human FT organoids (derived from epithelial progenitors) that presented ciliated and secretory cells, maintained stemness, and responded to reproductive hormones [173]. Later, Rose et al. discovered that organoids from the distal section produced larger spheroids and also established aldehyde dehydrogenase (ADLH) as a biomarker for FT organoid formation [174]. FT organoids have also been cultured in a 3D thermo-reversible gelation polymer (TGP), having conserved their putative markers [175].

Regarding oviductal pathology, FT epithelial cells were recently linked to the etiology of HGSOC and were found to be negatively impacted during episodes of pelvic inflammatory disease (PID) [172]. Xie et al. derived the first cancerous FT organoids in 2018 from the conditional transformation of related protein 53 (Trp53) and BRCA1 mutant mice [16]. Later, Zhang et al. engineered murine FT organoids with CRISPR/Cas9 technology to model the different combinations of mutations presented by HGSOC patients and to develop platforms to test personalized chemotherapy regimens [176]. To model PID, Yu et al. infected FT organoids with two common vaginal bacteria species and reported the expression of acute inflammation markers [177].

Interestingly, Chang et al. co-cultured FT organoids with mesenchymal FT stem cells and umbilical endothelial cells, resulting in more complex in vitro models. These FT assembloids combined different cell types, representing a new model for studying the regeneration and malignant transformation of the tubal epithelium [178].

### 6.3. Endometrium

The endometrium, the innermost layer of the uterus where the embryo is implanted following fertilization, is constituted by a layer of columnar secretory and ciliated epithelium, along with numerous tubular glands, and is supported by an underlying stromal component. In human females, the endometrium undergoes cyclical phases of shedding, regeneration, and differentiation every month [179].

Two independent groups developed endometrial organoids from human and mouse endometrial biopsies in 2017 [22,73]. These organoids were derived from the endometrial epithelium and mimicked the biological features of the epithelial glands. Specifically, they developed a void lumen, maintained apico-basal polarity, and were correctly divided through different passages [179]. Moreover, the human endometrial organoids (hEOs) were differentiated into secretory and gestational hEOs, which, respectively, mimicked the proliferative and gestational phases of the menstrual cycle by overexpressing the SPP1, PAEP, LIF, and 17HSDβ2 implantation markers [22]. In 2019, Haider et al. showed that the hEO’s ciliated phenotype could be induced by the coordinated action of estrogen and NOTCH signaling during the proliferative phase [180]. Notably, Francés-Herrero et al. reported how supplementing the hEO culture media with hydrogels derived from decellularized porcine endometrium improved cell proliferation, preserved long-term stability, and maintained stemness properties [23]. Elsewhere, hEOs were successfully cultured in pure bovine and human endometrial-derived hydrogels (rather than in synthetic biomaterials) [181]. Intriguingly, menstrual blood is emerging as a non-invasive source of cells for hEOs [182]. These organoids demonstrated similar proliferation, phenotype, and gene signatures in comparison to hEOs derived from standard biopsies.

The hEOs used to model a broad spectrum of endometrial pathologies [183], including adenomyosis [184], endometriotic lesions [15,185], and endometrial carcinoma [186,187,188,189], have successfully recapitulated the corresponding disease traits and served to unveil novel biomarkers. For instance, Juárez-Barber et al. demonstrated that adenomyosis organoids overexpressed TGF-β2 and SMAD3 compared to healthy endometrial organoids [184]. Furthermore, endometriosis organoids have revealed the epigenetic mechanisms that underlie endometriosis, as these organoids preserve the methylation levels of ectopic endometrial lesions [185]. Elsewhere, Jamaluddin et al. created patient-derived endometrial carcinoma organoids from 20 patients to characterize intra-tumoral proteomic differences [188]; understanding tumor heterogeneity is crucial for developing targeted life-saving cancer therapies.

Notably, hEOs co-cultured with stromal components (assembloids) offer a superior resemblance to the native endometrium, and the addition of embryos offers a realistic insight into implantation processes [190]. Despite the advantage of containing two different cell types, endometrial assembloid models have not yet been able to achieve propagation in vitro [19,191]. Therefore, hEOs remain the gold standard for studying human endometrial physiology.

### 6.4. Cervix

The cervix acts as a barrier between the vagina and uterus, producing mucus that either facilitates or obstructs the passage of sperm, depending on the stage of the menstrual cycle [192]. The cervix is divided into two distinct regions: the ectocervix, formed by stratified squamous epithelium, and the endocervix, composed of columnar epithelium [193].

Long-term, expandable cervical organoid models were successfully derived from both the stratified squamous ectocervix and the columnar endocervix [194]. Whether derived from tissues of mouse or human origin, these organoids demonstrated remarkable similarity to the respective in vivo tissues, faithfully replicating the architectural arrangement and phenotypic characteristics [194].

Regarding cervical pathologies, Chumduri et al. employed cervical organoids to study how cervical epithelial junction integrity prevents the emergence of metaplasia. The transcriptomic analysis of cervical organoids derived from healthy tissue versus those from malignant tissue revealed shared expression profiles between endocervical organoids and adenocarcinomas and between ectocervical organoids and squamous cell carcinomas. Based on their observations, this group postulated that the carcinomas arose from malignant transformations in diverse cell lineages [194]. In the same year, another group established human ecto- and endocervical 3D organoids that stably recapitulated physiological and carcinogenic traits, growing as xenografts in mice [195].

### 6.5. Vagina

The vagina is a tube-like structure composed of fibrous and muscular tissue connecting the cervix to the external genitalia. It is the channel through which uterine secretions are expelled and serves as the entryway for the penis and the fetal pathway during childbirth [196]. Like the ectocervix, the mucosa of the vagina is lined with stratified squamous epithelium.

Currently, there is no existing account of human vaginal organoids. The scientific literature only describes the establishment and sustained cultivation of murine vaginal organoids [197] that resembled the in vivo architecture of vaginal tissue, with a stratified squamous epithelial arrangement and tumor protein P63-positive cells along the periphery. The viability of vaginal organoids hinges on the intrinsic Wnt signaling pathway, which governs both their proliferation and differentiation [197]. While increasing Wnt levels in the culture medium improved organoid growth, exceedingly high Wnt concentrations suppressed growth, highlighting the importance of Wnt regulation in vaginal epithelial cells. This innovative model is poised to play a pivotal role in discerning the intricate mechanisms underlying the regeneration and equilibrium of the vaginal epithelium [197].

### 6.6. Future Challenges in Modeling the Female Reproductive Tract

The development of 3D in vitro culture systems that maintain the original characteristics of the distinct tissues and organs within the female reproductive tract has progressed substantially from 2017, when two separate groups described the first human endometrial organoids [22,73], to 2021, when Rawlings et al. introduced assembloid models combining multiple cell types in the same 3D culture [19]. Nevertheless, static 3D culture still has several limitations, such as the lack of mechanical flow dynamics, poor connectivity with other cell culture wells, challenging pH and temperature standardization, and the elimination of accumulated toxic metabolites [198]. Microfluidic devices have begun to address these limitations, using pumps to dynamically circulate the culture medium between connected cell chambers to constantly renew nutrients while simultaneously removing toxic metabolites [199]. Indeed, the shared secretome in microfluidic systems proved essential for modeling realistic cell–cell communication, tissue development, and immune responses [200]. These advances position microfluidics as another emerging bioengineering approach to enhance in vitro culture systems [183].

To date, EVATAR is the most comprehensive microfluidic platform to model the endocrine interactions occurring in the human female reproductive tract and peripheral tissues during the menstrual cycle and pregnancy [201]. This sophisticated model replicates the in vivo dynamics of the 28-day menstrual cycle by continuously circulating media between units that represent the ovary, FT, uterus, cervix, and liver. However, EVATAR requires large samples of primary tissues to construct each chip—potentially implying the need for invasive biopsies and poor scalability—thereby restricting in vitro propagation and the feasibility of biobanking [201].

Combining organoids derived from the human female reproductive tract within microfluidic platforms could overcome these limitations and potentially elucidate the mechanisms underlying infertility and gynecological disorders. Such platforms could be used to generate in vitro models of decidualization, implantation, placentation, and maternal–fetal crosstalk during fetal development [202]. Similarly, this technology could be applied to model, investigate, and pharmacologically modulate the pathophysiology of intractable diseases such as ovarian cancer and endometriosis [202]. The in vitro simulation of these diseases could enhance drug testing efficiency and the exploration of individualized therapeutic approaches.

## 7. Conclusions

In conclusion, organoid models have revolutionized tissue engineering, primarily due to their ability to faithfully reproduce the biological characteristics of the native tissue when compared to conventional in vitro approaches. Although the precise definition of the term organoid remains controversial, we defined them for this article as an aggregation of a single type of organ-specific cells embedded in an extracellular scaffold (commonly hydrogels) and cultivated in a defined culture medium. Organoids are applied in various biomedical disciplines, including reproductive medicine, and contribute significantly to biobanking, disease modeling, drug testing, and personalized medicine. These applications have proved invaluable to advancing understanding of the female reproductive tract, studying various gynecological pathologies and/or the associated causes of infertility, discovering novel biomarkers, and ultimately, developing more effective and personalized treatments for affected patients.

## Data Availability

Not applicable.

## References

[1] Clevers H. (2016). Modeling Development and Disease with Organoids. Cell.

[2] Zhao Z., Chen X., Dowbaj A.M., Sljukic A., Bratlie K., Lin L., Fong E.L.S., Balachander G.M., Chen Z., Soragni A. (2022). Organoids. Nat. Rev. Methods Prim..

[3] Heydari Z., Moeinvaziri F., Agarwal T., Pooyan P., Shpichka A., Maiti T.K., Timashev P., Baharvand H., Vosough M. (2021). Organoids: A Novel Modality in Disease Modeling. Bio-Des. Manuf..

[4] Mu P., Zhou S., Lv T., Xia F., Shen L., Wan J., Wang Y., Zhang H., Cai S., Peng J. (2023). Newly Developed 3D in Vitro Models to Study Tumor–Immune Interaction. J. Exp. Clin. Cancer Res..

[5] Gjorevski N., Nikolaev M., Brown T.E., Mitrofanova O., Brandenberg N., DelRio F.W., Yavitt F.M., Liberali P., Anseth K.S., Lutolf M.P. (2022). Tissue Geometry Drives Deterministic Organoid Patterning. Science.

[6] Rossi G., Manfrin A., Lutolf M.P. (2018). Progress and Potential in Organoid Research. Nat. Rev. Genet..

[7] Petrovic V., Fallon J., Kuester F. (2007). Visualizing Whole-Brain DTI Tractography with GPU-Based Tuboids and LoD Management. IEEE Trans. Vis. Comput. Graph..

[8] Sanaki-Matsumiya M., Matsuda M., Gritti N., Nakaki F., Sharpe J., Trivedi V., Ebisuya M. (2022). Periodic Formation of Epithelial Somites from Human Pluripotent Stem Cells. Nat. Commun..

[9] Hofer M., Lutolf M.P. (2021). Engineering Organoids. Nat. Rev. Mater..

[10] Tauro B.J., Greening D.W., Mathias R.A., Mathivanan S., Ji H., Simpson R.J. (2013). Two Distinct Populations of Exosomes Are Released from LIM1863 Colon Carcinoma Cell-Derived Organoids. Mol. Cell. Proteom..

[11] Fennema E., Rivron N., Rouwkema J., van Blitterswijk C., De Boer J. (2013). Spheroid Culture as a Tool for Creating 3D Complex Tissues. Trends Biotechnol..

[12] Jeppesen M., Hagel G., Glenthoj A., Vainer B., Ibsen P., Harling H., Thastrup O., Jørgensen L.N., Thastrup J. (2017). Short-Term Spheroid Culture of Primary Colorectal Cancer Cells as an in Vitro Model for Personalizing Cancer Medicine. PLoS ONE.

[13] Luan Q., Becker J.H., Macaraniag C., Massad M.G., Zhou J., Shimamura T., Papautsky I. (2022). Non-Small Cell Lung Carcinoma Spheroid Models in Agarose Microwells for Drug Response Studies. Lab Chip.

[14] Kopper O., de Witte C.J., Lõhmussaar K., Valle-Inclan J.E., Hami N., Kester L., Balgobind A.V., Korving J., Proost N., Begthel H. (2019). An Organoid Platform for Ovarian Cancer Captures Intra- and Interpatient Heterogeneity. Nat. Med..

[15] Boretto M., Maenhoudt N., Luo X., Hennes A., Boeckx B., Bui B., Heremans R., Perneel L., Kobayashi H., Van Zundert I. (2019). Patient-Derived Organoids from Endometrial Disease Capture Clinical Heterogeneity and Are Amenable to Drug Screening. Nat. Cell Biol..

[16] Xie Y., Park E.S., Xiang D., Li Z. (2018). Long-Term Organoid Culture Reveals Enrichment of Organoid-Forming Epithelial Cells in the Fimbrial Portion of Mouse Fallopian Tube. Stem Cell Res..

[17] Schutgens F., Clevers H. (2020). Human Organoids: Tools for Understanding Biology and Treating Diseases. Annu. Rev. Pathol. Mech. Dis..

[18] Kim E., Choi S., Kang B., Kong J.H., Kim Y., Yoon W.H., Lee H.R., Kim S.E., Kim H.M., Lee H.S. (2020). Creation of Bladder Assembloids Mimicking Tissue Regeneration and Cancer. Nature.

[19] Rawlings T.M., Makwana K., Taylor D.M., Molè M.A., Fishwick K.J., Tryfonos M., Odendaal J., Hawkes A., Zernicka-Goetz M., Hartshorne G.M. (2021). Modelling the Impact of Decidual Senescence on Embryo Implantation in Human Endometrial Assembloids. eLife.

[20] Xue J., Sun N., Liu Y. (2022). Self-Assembled Nano-Peptide Hydrogels with Human Umbilical Cord Mesenchymal Stem Cell Spheroids Accelerate Diabetic Skin Wound Healing by Inhibiting Inflammation and Promoting Angiogenesis. Int. J. Nanomed..

[21] Moroni L., Elisseeff J.H. (2008). Biomaterials Engineered for Integration. Mater. Today.

[22] Turco M.Y., Gardner L., Hughes J., Cindrova-Davies T., Gomez M.J., Farrell L., Hollinshead M., Marsh S.G.E., Brosens J.J., Critchley H.O. (2017). Long-Term, Hormone-Responsive Organoid Cultures of Human Endometrium in a Chemically Defined Medium. Nat. Cell Biol..

[23] Francés-Herrero E., Juárez-Barber E., Campo H., López-Martínez S., de Miguel-Gómez L., Faus A., Pellicer A., Ferrero H., Cervelló I. (2021). Improved Models of Human Endometrial Organoids Based on Hydrogels from Decellularized Endometrium. J. Pers. Med..

[24] Du Z., Zang Z., Luo J., Liu T., Yang L., Cai Y., Wang L., Zhang D., Zhao J., Gao J. (2023). Chronic Exposure to (2R,6R)-Hydroxynorketamine Induces Developmental Neurotoxicity in HESC-Derived Cerebral Organoids. J. Hazard. Mater..

[25] Pitstick A.L., Poling H.M., Sundaram N., Lewis P.L., Kechele D.O., Sanchez J.G., Scott M.A., Broda T.R., Helmrath M.A., Wells J.M. (2022). Aggregation of Cryopreserved Mid-Hindgut Endoderm for More Reliable and Reproducible HPSC-Derived Small Intestinal Organoid Generation. Stem Cell Rep..

[26] Deng H. (2015). Derivation of Pluripotent Stem Cells with in Vivo Embryonic and Extraembryonic Potency. Cell.

[27] Tsuruta S., Uchida H., Akutsu H. (2020). Intestinal Organoids Generated from Human Pluripotent Stem Cells. JMA J..

[28] Thomson J.A., Itskovitz-eldor J., Shapiro S.S., Michelle A., Swiergiel J.J., Marshall V.S., Jones J.M., Thomson J.A., Itskovitz-eldor J., Shapiro S.S. (1998). Embryonic Stem Cell Lines Derived from Human Blastocysts. Science.

[29] Takahashi K., Yamanaka S. (2006). Induction of Pluripotent Stem Cells from Mouse Embryonic and Adult Fibroblast Cultures by Defined Factors. Cell.

[30] Kakni P., López-Iglesias C., Truckenmüller R., Habibović P., Giselbrecht S. (2022). Reversing Epithelial Polarity in Pluripotent Stem Cell-Derived Intestinal Organoids. Front. Bioeng. Biotechnol..

[31] Rempel S.K., Welch M.J., Ludwig A.L., Phillips M.J., Kancherla Y., Zack D.J., Gamm D.M., Gómez T.M. (2022). Human photoreceptors switch from autonomous axon extension to cell-mediated process pulling during synaptic marker redistribution. Cell Rep..

[32] Suhito I.R., Kim J.W., Koo K.M., Nam S.A., Kim Y.K., Kim T.H. (2022). In Situ Detection of Kidney Organoid Generation From Stem Cells Using a Simple Electrochemical Method. Adv. Sci..

[33] Van Lent J., Vendredy L., Adriaenssens E., Da Silva Authier T., Asselbergh B., Kaji M., Weckhuysen S., Van Den Bosch L., Baets J., Timmerman V. (2022). Downregulation of PMP22 Ameliorates Myelin Defects in IPSC-Derived Human Organoid Cultures of CMT1A. Brain.

[34] Ning R., Zheng D., Xie B., Gao G., Xu J., Xu P., Wang Y., Peng F., Jiang B., Ge J. (2022). Spatial and Temporal Development of Müller Glial Cells in HiPSC-Derived Retinal Organoids Facilitates the Cell Enrichment and Transcriptome Analysis. Front. Cell. Neurosci..

[35] Shinozawa T., Kimura M., Cai Y., Saiki N., Yoneyama Y., Ouchi R., Koike H., Maezawa M., Zhang R.R., Dunn A. (2021). High-Fidelity Drug-Induced Liver Injury Screen Using Human Pluripotent Stem Cell–Derived Organoids. Gastroenterology.

[36] Lyu Q., Gong S., Lees J.G., Yin J., Yap L.W., Kong A.M., Shi Q., Fu R., Zhu Q., Dyer A. (2022). A Soft and Ultrasensitive Force Sensing Diaphragm for Probing Cardiac Organoids Instantaneously and Wirelessly. Nat. Commun..

[37] Ma J., Wang N.Y., Jagani R., Wang H.S. (2023). Proarrhythmic Toxicity of Low Dose Bisphenol A and Its Analogs in Human IPSC-Derived Cardiomyocytes and Human Cardiac Organoids through Delay of Cardiac Repolarization. Chemosphere.

[38] Hayal T.B., Doğan A. (2022). Feeder-Free Human Embryonic Stem Cell Culture Under Defined Culture Conditions. Methods Mol. Biol..

[39] Kurosawa H. (2007). Methods for Inducing Embryoid Body Formation: In Vitro Differentiation System of Embryonic Stem Cells. J. Biosci. Bioeng..

[40] Watanabe K., Ueno M., Kamiya D., Nishiyama A., Matsumura M., Wataya T., Takahashi J.B., Nishikawa S., Nishikawa S.I., Muguruma K. (2007). A ROCK Inhibitor Permits Survival of Dissociated Human Embryonic Stem Cells. Nat. Biotechnol..

[41] Atanasova V.S., de Jesus Cardona C., Hejret V., Tiefenbacher A., Mair T., Tran L., Pfneissl J., Draganić K., Binder C., Kabiljo J. (2023). Mimicking Tumor Cell Heterogeneity of Colorectal Cancer in a Patient-Derived Organoid-Fibroblast Model. Cell. Mol. Gastroenterol. Hepatol..

[42] Kageyama T., Miyata H., Seo J., Nanmo A., Fukuda J. (2023). In Vitro Hair Follicle Growth Model for Drug Testing. Sci. Rep..

[43] Minoli M., Cantore T., Hanhart D., Kiener M., Fedrizzi T., La Manna F., Karkampouna S., Chouvardas P., Genitsch V., Rodriguez-Calero A. (2023). Bladder Cancer Organoids as a Functional System to Model Different Disease Stages and Therapy Response. Nat. Commun..

[44] Pan M., Xiao T., Xu L., Xie Y., Ge W. (2023). UTP18-Mediated P21 MRNA Instability Drives Adenoma-Carcinoma Progression in Colorectal Cancer. Cell Rep..

[45] Zhou H., Zhang Q., Huang W., Zhou S., Wang Y., Zeng X., Wang H., Xie W., Kong H. (2023). NLRP3 Inflammasome Mediates Silica-Induced Lung Epithelial Injury and Aberrant Regeneration in Lung Stem/Progenitor Cell-Derived Organotypic Models. Int. J. Biol. Sci..

[46] D’Imprima E., Garcia Montero M., Gawrzak S., Ronchi P., Zagoriy I., Schwab Y., Jechlinger M., Mahamid J. (2023). Light and Electron Microscopy Continuum-Resolution Imaging of 3D Cell Cultures. Dev. Cell.

[47] Hu P., Zhang W., Xin H., Deng G. (2016). Single Cell Isolation and Analysis. Front. Cell Dev. Biol..

[48] Aronowitz J.A., Lockhart R.A., Hakakian C.S. (2015). Mechanical versus Enzymatic Isolation of Stromal Vascular Fraction Cells from Adipose Tissue. SpringerPlus.

[49] Reddy P., Zhao D., Ravikumar V., Lauder E., Li L., Sun Y., Oravecz-Wilson K., Brooks M., Keller E., Chen F. (2023). Inflammatory Memory Restrains Intestinal Stem Cell Regeneration. Res. Sq..

[50] Arutyunyan A., Roberts K., Troulé K., Wong F.C.K., Sheridan M.A., Kats I., Garcia-Alonso L., Velten B., Hoo R., Ruiz-Morales E.R. (2023). Spatial Multiomics Map of Trophoblast Development in Early Pregnancy. Nature.

[51] Wang Y., Chiola S., Yang G., Russell C., Armstrong C.J., Wu Y., Spampanato J., Tarboton P., Ullah H.M.A., Edgar N.U. (2022). Modeling Human Telencephalic Development and Autism-Associated SHANK3 Deficiency Using Organoids Generated from Single Neural Rosettes. Nat. Commun..

[52] Hendriks D., Brouwers J.F., Hamer K., Geurts M.H., Luciana L., Massalini S., López-Iglesias C., Peters P.J., Rodríguez-Colman M.J., Chuva de Sousa Lopes S. (2023). Engineered Human Hepatocyte Organoids Enable CRISPR-Based Target Discovery and Drug Screening for Steatosis. Nat. Biotechnol..

[53] Fischer J., Fernández Ortuño E., Marsoner F., Artioli A., Peters J., Namba T., Eugster Oegema C., Huttner W.B., Ladewig J., Heide M. (2022). Human-specific ARHGAP11B Ensures Human-like Basal Progenitor Levels in Hominid Cerebral Organoids. EMBO Rep..

[54] Bartsch D., Kalamkar K., Ahuja G., Lackmann J.W., Hescheler J., Weber T., Bazzi H., Clamer M., Mendjan S., Papantonis A. (2023). MRNA Translational Specialization by RBPMS Presets the Competence for Cardiac Commitment in HESCs. Sci. Adv..

[55] Kwilas A.R., Donahue R.N., Tsang K.Y., Hodge J.W. (2015). Immune consequences of tyrosine kinase inhibitors that synergize with cancer immunotherapy. Cancer Cell Microenviron..

[56] Dutta D., Heo I., Clevers H. (2017). Disease Modeling in Stem Cell-Derived 3D Organoid Systems. Trends Mol. Med..

[57] Engler A.J., Sen S., Sweeney H.L., Discher D.E. (2006). Matrix Elasticity Directs Stem Cell Lineage Specification. Cell.

[58] Ho T.C., Chang C.C., Chan H.P., Chung T.W., Shu C.W., Chuang K.P., Duh T.H., Yang M.H., Tyan Y.C. (2022). Hydrogels: Properties and Applications in Biomedicine. Molecules.

[59] Özmert E., Arslan U. (2020). Management of Retinitis Pigmentosa by Wharton’s Jelly-Derived Mesenchymal Stem Cells: Prospective Analysis of 1-Year Results. Stem Cell Res. Ther..

[60] Skardal A., Shupe T., Atala A. (2016). Organoid-on-a-Chip and Body-on-a-Chip Systems for Drug Screening and Disease Modeling. Drug Discov. Today.

[61] Sun J., Zhang Y., Li B., Gu Y., Chen L. (2017). Controlled Release of BMP-2 from a Collagen-Mimetic Peptide-Modified Silk Fibroin–Nanohydroxyapatite Scaffold for Bone Regeneration. J. Mater. Chem. B.

[62] Ning L., Malmström H., Ren Y.F. (2015). Porous Collagen-Hydroxyapatite Scaffolds with Mesenchymal Stem Cells for Bone Regeneration. J. Oral Implantol..

[63] Wiwatpanit T., Murphy A.R., Lu Z., Urbanek M., Burdette J.E., Woodruff T.K., Kim J.J. (2020). Scaffold-Free Endometrial Organoids Respond to Excess Androgens Associated with Polycystic Ovarian Syndrome. J. Clin. Endocrinol. Metab..

[64] Gomez-Florit M., Pardo A., Domingues R.M.A., Graça A.L., Babo P.S., Reis R.L., Gomes M.E. (2020). Natural-Based Hydrogels for Tissue Engineering Applications. Molecules.

[65] Davari N., Bakhtiary N., Khajehmohammadi M., Sarkari S., Tolabi H., Ghorbani F., Ghalandari B. (2022). Protein-Based Hydrogels: Promising Materials for Tissue Engineering. Polymers.

[66] Passaniti A., Kleinman H.K., Martin G.R. (2022). Matrigel: History/Background, Uses, and Future Applications. J. Cell Commun. Signal..

[67] Kashfi H., Jinks N., Nateri A.S. (2020). Generating and Utilizing Murine Cas9-Expressing Intestinal Organoids for Large-Scale Knockout Genetic Screening. Methods Mol. Biol..

[68] Kulkeaw K., Tubsuwan A., Tongkrajang N., Whangviboonkij N. (2020). Generation of Human Liver Organoids from Pluripotent Stem Cell-Derived Hepatic Endoderms. PeerJ.

[69] Yu C., Kang R., Tang D. (2023). Organoids Models of Pancreatic Duct Adenocarcinoma. Methods Mol. Biol..

[70] Maru Y., Tanaka N., Itami M., Hippo Y. (2019). Efficient Use of Patient-Derived Organoids as a Preclinical Model for Gynecologic Tumors. Gynecol. Oncol..

[71] Ma L., Li J., Nie Q., Zhang Q., Liu S., Ge D., You Z. (2017). Organoid Culture of Human Prostate Cancer Cell Lines LNCaP and C4-2B. Am. J. Clin. Exp. Urol..

[72] Drost J., Karthaus W.R., Gao D., Driehuis E., Sawyers C.L., Chen Y., Clevers H. (2016). Organoid Culture Systems for Prostate Epithelial and Cancer Tissue. Nat. Protoc..

[73] Boretto M., Cox B., Noben M., Hendriks N., Fassbender A., Roose H., Amant F., Timmerman D., Tomassetti C., Vanhie A. (2017). Development of Organoids from Mouse and Human Endometrium Showing Endometrial Epithelium Physiology and Long-Term Expandability. Development.

[74] Fatehullah A., Tan S.H., Barker N. (2016). Organoids as an in Vitro Model of Human Development and Disease. Nat. Cell Biol..

[75] Huch M., Dorrell C., Boj S.F., Van Es J.H., Li V.S.W., Van De Wetering M., Sato T., Hamer K., Sasaki N., Finegold M.J. (2013). In Vitro Expansion of Single Lgr5+ Liver Stem Cells Induced by Wnt-Driven Regeneration. Nature.

[76] Miller A.J., Dye B.R., Ferrer-Torres D., Hill D.R., Overeem A.W., Shea L.D., Spence J.R. (2019). Generation of Lung Organoids from Human Pluripotent Stem Cells in Vitro. Nat. Protoc..

[77] Lancaster M.A., Renner M., Martin C.A., Wenzel D., Bicknell L.S., Hurles M.E., Homfray T., Penninger J.M., Jackson A.P., Knoblich J.A. (2013). Cerebral Organoids Model Human Brain Development and Microcephaly. Nature.

[78] Takebe T., Sekine K., Enomura M., Koike H., Kimura M., Ogaeri T., Zhang R.R., Ueno Y., Zheng Y.W., Koike N. (2013). Vascularized and Functional Human Liver from an IPSC-Derived Organ Bud Transplant. Nature.

[79] Noori A., Ashrafi S.J., Vaez-Ghaemi R., Hatamian-Zaremi A., Webster T.J. (2017). A Review of Fibrin and Fibrin Composites for Bone Tissue Engineering. Int. J. Nanomed..

[80] Tang-Schomer M.D., Wu W.B., Kaplan D.L., Bookland M.J. (2018). In Vitro 3D Regeneration-like Growth of Human Patient Brain Tissue. J. Tissue Eng. Regen. Med..

[81] Cardenas D., Bhalchandra S., Lamisere H., Chen Y., Zeng X.L., Ramani S., Karandikar U.C., Kaplan D.L., Estes M.K., Ward H.D. (2020). Two- and Three-Dimensional Bioengineered Human Intestinal Tissue Models for Cryptosporidium. Methods Mol. Biol..

[82] Li G., Sun S. (2022). Silk Fibroin-Based Biomaterials for Tissue Engineering Applications. Molecules.

[83] Chooi W.H., Ng C.Y., Ow V., Harley J., Ng W., Hor J.H., Low K.E., Malleret B., Xue K., Ng S.Y. (2023). Defined Alginate Hydrogels Support Spinal Cord Organoid Derivation, Maturation, and Modeling of Spinal Cord Diseases. Adv. Healthc. Mater..

[84] Zakhem E., Raghavan S., Gilmont R.R., Bitar K.N. (2012). Chitosan-Based Scaffolds for the Support of Smooth Muscle Constructs in Intestinal Tissue Engineering. Biomaterials.

[85] Krüger M., Oosterhoff L.A., van Wolferen M.E., Schiele S.A., Walther A., Geijsen N., De Laporte L., van der Laan L.J.W., Kock L.M., Spee B. (2020). Cellulose Nanofibril Hydrogel Promotes Hepatic Differentiation of Human Liver Organoids. Adv. Healthc. Mater..

[86] Lee K.Y., Mooney D.J. (2012). Alginate: Properties and Biomedical Applications. Prog. Polym. Sci..

[87] Cakir B., Xiang Y., Tanaka Y., Kural M.H., Parent M., Kang Y.J., Chapeton K., Patterson B., Yuan Y., He C.S. (2019). Engineering of Human Brain Organoids with a Functional Vascular-like System. Nat. Methods.

[88] Zhao J., Qiu P., Wang Y., Wang Y., Zhou J., Zhang B., Zhang L., Gou D. (2023). Chitosan-Based Hydrogel Wound Dressing: From Mechanism to Applications, a Review. Int. J. Biol. Macromol..

[89] Davoudi Z., Peroutka-Bigus N., Bellaire B., Jergens A., Wannemuehler M., Wang Q. (2021). Gut Organoid as a New Platform to Study Alginate and Chitosan Mediated PLGA Nanoparticles for Drug Delivery. Mar. Drugs.

[90] Giobbe G.G., Crowley C., Luni C., Campinoti S., Khedr M., Kretzschmar K., De Santis M.M., Zambaiti E., Michielin F., Meran L. (2019). Extracellular Matrix Hydrogel Derived from Decellularized Tissues Enables Endodermal Organoid Culture. Nat. Commun..

[91] Kim J.W., Nam S.A., Yi J., Kim J.Y., Lee J.Y., Park S.Y., Sen T., Choi Y.M., Lee J.Y., Kim H.L. (2022). Kidney Decellularized Extracellular Matrix Enhanced the Vascularization and Maturation of Human Kidney Organoids. Adv. Sci..

[92] De Hilster R.H.J., Sharma P.K., Jonker M.R., White E.S., Gercama E.A., Roobeek M., Timens W., Harmsen M.C., Hylkema M.N., Burgess J.K. (2020). Human Lung Extracellular Matrix Hydrogels Resemble the Stiffness and Viscoelasticity of Native Lung Tissue. Am. J. Physiol. Lung Cell. Mol. Physiol..

[93] Hussein K.H., Park K.M., Yu L., Kwak H.H., Woo H.M. (2020). Decellularized Hepatic Extracellular Matrix Hydrogel Attenuates Hepatic Stellate Cell Activation and Liver Fibrosis. Mater. Sci. Eng. C Mater. Biol. Appl..

[94] Simsa R., Rothenbücher T., Gürbüz H., Ghosheh N., Emneus J., Jenndahl L., Kaplan D.L., Bergh N., Serrano A.M., Fogelstrand P. (2021). Brain Organoid Formation on Decellularized Porcine Brain ECM Hydrogels. PLoS ONE.

[95] Zheng J., Liu Y., Hou C., Li Z., Yang S., Liang X., Zhou L., Guo J., Zhang J., Huang X. (2022). Ovary-Derived Decellularized Extracellular Matrix-Based Bioink for Fabricating 3D Primary Ovarian Cells-Laden Structures for Mouse Ovarian Failure Correction. Int. J. Bioprint..

[96] Crapo P.M., Gilbert T.W., Badylak S.F. (2011). An Overview of Tissue and Whole Organ Decellularization Processes. Biomaterials.

[97] Gazia C., Tamburrini R., Asthana A., Chaimov D., Muir S.M., Marino D.I., Delbono L., Villani V., Perin L., Di Nardo P. (2019). Extracellular Matrix-Based Hydrogels Obtained from Human Tissues: A Work Still in Progress. Curr. Opin. Organ Transplant..

[98] Pérez R.A., Won J.E., Knowles J.C., Kim H.W. (2013). Naturally and Synthetic Smart Composite Biomaterials for Tissue Regeneration. Adv. Drug Deliv. Rev..

[99] Place E.S., Evans N.D., Stevens M.M. (2009). Complexity in Biomaterials for Tissue Engineering. Nat. Mater..

[100] Tian C.-M., Yang M.-F., Xu H.-M., Zhu M.-Z., Yue N.-N., Zhang Y., Shi R.-Y., Yao J., Wang L.-S., Liang Y.-J. (2023). Stem Cell-Derived Intestinal Organoids: A Novel Modality for IBD. Cell Death Discov..

[101] Huch M., Gehart H., Van Boxtel R., Hamer K., Blokzijl F., Verstegen M.M.A., Ellis E., Van Wenum M., Fuchs S.A., De Ligt J. (2015). Long-Term Culture of Genome-Stable Bipotent Stem Cells from Adult Human Liver. Cell.

[102] Green R., Abidian M.R. (2015). Conducting Polymers for Neural Prosthetic and Neural Interface Applications. Adv. Mater..

[103] Wilson R.L., Swaminathan G., Ettayebi K., Bomidi C., Zeng X.L., Blutt S.E., Estes M.K., Grande-Allen K.J. (2021). Protein-Functionalized Poly(Ethylene Glycol) Hydrogels as Scaffolds for Monolayer Organoid Culture. Tissue Eng. Part C Methods.

[104] KarbalaeiMahdi A., Shahrousvand M., Javadi H.R., Ghollasi M., Norouz F., Kamali M., Salimi A. (2017). Neural Differentiation of Human Induced Pluripotent Stem Cells on Polycaprolactone/Gelatin Bi-Electrospun Nanofibers. Mater. Sci. Eng. C Mater. Biol. Appl..

[105] Zhang S. (2003). Fabrication of Novel Biomaterials through Molecular Self-Assembly. Nat. Biotechnol..

[106] Zhang Y.S., Khademhosseini A. (2017). Advances in Engineering Hydrogels. Science.

[107] Urbischek M., Rannikmae H., Foets T., Ravn K., Hyvönen M., de la Roche M. (2019). Organoid Culture Media Formulated with Growth Factors of Defined Cellular Activity. Sci. Rep..

[108] Asnaghi M.A., Smith T., Martin I., Wendt D. (2014). Bioreactors: Enabling Technologies for Research and Manufacturing.

[109] Yao T., Asayama Y. (2017). Animal-Cell Culture Media: History, Characteristics, and Current Issues. Reprod. Med. Biol..

[110] van der Valk J., Bieback K., Buta C., Cochrane B., Dirks W.G., Fu J., Hickman J.J., Hohensee C., Kolar R., Liebsch M. (2018). Fetal Bovine Serum (FBS): Past–Present–Future. ALTEX.

[111] Andrée B., Bela K., Horvath T., Lux M., Ramm R., Venturini L., Ciubotaru A., Zweigerdt R., Haverich A., Hilfiker A. (2014). Successful Re-Endothelialization of a Perfusable Biological Vascularized Matrix (BioVaM) for the Generation of 3D Artificial Cardiac Tissue. Basic Res. Cardiol..

[112] Kaneko T., LePage G.A., Shnitka T.K. (1980). KLN205—A Murine Lung Carcinoma Cell Line. In Vitro.

[113] Stacey G.N. (2011). Chapter 7 Cell Culture Contamination. Cancer Cell Culture. Methods in Molecular Biology.

[114] Ryu A.H., Eckalbar W.L., Kreimer A., Yosef N., Ahituv N. (2017). Use Antibiotics in Cell Culture with Caution: Genome-Wide Identification of Antibiotic-Induced Changes in Gene Expression and Regulation. Sci. Rep..

[115] Stone N.R.H., Bicanic T., Salim R., Hope W. (2016). Liposomal Amphotericin B (AmBisome^®^): A Review of the Pharmacokinetics, Pharmacodynamics, Clinical Experience and Future Directions. Drugs.

[116] Goonoo N., Bhaw-Luximon A. (2019). Mimicking Growth Factors: Role of Small Molecule Scaffold Additives in Promoting Tissue Regeneration and Repair. RSC Adv..

[117] Aloia L., McKie M.A., Vernaz G., Cordero-Espinoza L., Aleksieva N., van den Ameele J., Antonica F., Font-Cunill B., Raven A., Aiese Cigliano R. (2019). Epigenetic Remodelling Licences Adult Cholangiocytes for Organoid Formation and Liver Regeneration. Nat. Cell Biol..

[118] Shi X., Li Y., Yuan Q., Tang S., Guo S., Zhang Y., He J., Zhang X., Han M., Liu Z. (2022). Integrated Profiling of Human Pancreatic Cancer Organoids Reveals Chromatin Accessibility Features Associated with Drug Sensitivity. Nat. Commun..

[119] Chen H.Y., Swaroop M., Papal S., Mondal A.K., Song H.B., Campello L., Tawa G.J., Regent F., Shimada H., Nagashima K. (2023). Reserpine Maintains Photoreceptor Survival in Retinal Ciliopathy by Resolving Proteostasis Imbalance and Ciliogenesis Defects. eLife.

[120] Walaas G.A., Gopalakrishnan S., Bakke I., Skovdahl H.K., Flatberg A., Østvik A.E., Sandvik A.K., Bruland T. (2023). Physiological Hypoxia Improves Growth and Functional Differentiation of Human Intestinal Epithelial Organoids. Front. Immunol..

[121] Bunney P.E., Zink A.N., Holm A.A., Billington C.J., Kotz C.M. (2017). Orexin activation counteracts decreases in nonexercise activity thermogenesis (NEAT) caused by high-fat diet. Physiol. Behav..

[122] Boj S.F., Hwang C.-I., Baker L.A., Chio I.I.C., Engle D.D., Corbo V., Jager M., Ponz-sarvise M., Tiriac H., Spector M.S. (2016). Organoid Model of Human and Mouse Pancreatic Ductal Adenocarcinoma. Cell.

[123] Below C.R., Kelly J., Brown A., Humphries J.D., Hutton C., Xu J., Lee B.Y., Cintas C., Zhang X., Stockdale L. (2022). A Microenvironment-Inspired Synthetic Three-Dimensional Model for Pancreatic Ductal Adenocarcinoma Organoids. Nat. Mater..

[124] Takasato M., Er P.X., Chiu H.S., Maier B., Baillie G.J., Ferguson C., Parton R.G., Wolvetang E.J., Roost M.S., De Sousa Lopes S.M.C. (2015). Kidney Organoids from Human IPS Cells Contain Multiple Lineages and Model Human Nephrogenesis. Nature.

[125] Kim S.Y., Kim S.M., Lim S., Lee J.Y., Choi S.J., Yang S.D., Yun M.R., Kim C.G., Gu S.R., Park C. (2021). Modeling Clinical Responses to Targeted Therapies by Patient-Derived Organoids of Advanced Lung Adenocarcinoma. Clin. Cancer Res..

[126] Saha A., Capowski E., Zepeda M.A.F., Nelson E.C., Gamm D.M., Sinha R. (2023). The primate fovea: Structure, function and development. Prog. Retin. Eye Res..

[127] Kim M., Mun H., Sung C.O., Cho E.J., Jeon H.J., Chun S.M., Jung D.J., Shin T.H., Jeong G.S., Kim D.K. (2019). Patient-Derived Lung Cancer Organoids as in Vitro Cancer Models for Therapeutic Screening. Nat. Commun..

[128] Baden P., Perez M.J., Raji H., Bertoli F., Kalb S., Illescas M., Spanos F., Giuliano C., Calogero A.M., Oldrati M. (2023). Glucocerebrosidase Is Imported into Mitochondria and Preserves Complex I Integrity and Energy Metabolism. Nat. Commun..

[129] Fujii M., Matano M., Toshimitsu K., Takano A., Mikami Y., Nishikori S., Sugimoto S., Sato T. (2018). Human Intestinal Organoids Maintain Self-Renewal Capacity and Cellular Diversity in Niche-Inspired Culture Condition. Cell Stem Cell.

[130] Jiang S., Xu F., Jin M., Wang Z., Xu X., Zhou Y., Wang J., Gu L., Fan H., Fan Y. (2023). Development of a High-Throughput Micropatterned Agarose Scaffold for Consistent and Reproducible HPSC-Derived Liver Organoids. Biofabrication.

[131] Lewis-Israeli Y.R., Wasserman A.H., Gabalski M.A., Volmert B.D., Ming Y., Ball K.A., Yang W., Zou J., Ni G., Pajares N. (2021). Self-Assembling Human Heart Organoids for the Modeling of Cardiac Development and Congenital Heart Disease. Nat. Commun..

[132] Finkbeiner C., Ortuño-Lizarán I., Sridhar A., Hooper M., Petter S., Reh T.A. (2022). Single-Cell ATAC-Seq of Fetal Human Retina and Stem-Cell-Derived Retinal Organoids Shows Changing Chromatin Landscapes during Cell Fate Acquisition. Cell Rep..

[133] Rodriguez-Gatica J.E., Iefremova V., Sokhranyaeva L., Yeung S.W.C.A., Breitkreuz Y., Brüstle O., Schwarz M.K., Kubitscheck U. (2022). Imaging Three-Dimensional Brain Organoid Architecture from Meso- to Nanoscale across Development. Development.

[134] Wang H.M., Zhang C.Y., Peng K.C., Chen Z.X., Su J.W., Li Y.F., Li W.F., Gao Q.Y., Zhang S.L., Chen Y.Q. (2023). Using Patient-Derived Organoids to Predict Locally Advanced or Metastatic Lung Cancer Tumor Response: A Real-World Study. Cell Rep. Med..

[135] Wang R., Kang N., Zhang W., Chen B., Xu S., Wu L. (2023). The Developmental Toxicity of PM2.5 on the Early Stages of Fetal Lung with Human Lung Bud Tip Progenitor Organoids. Environ. Pollut..

[136] Rockel A.F., Wagner N., Spenger P., Ergün S., Wörsdörfer P. (2023). Neuro-Mesodermal Assembloids Recapitulate Aspects of Peripheral Nervous System Development in Vitro. Stem Cell Rep..

[137] Silva A.C., Matthys O.B., Joy D.A., Kauss M.A., Natarajan V., Lai M.H., Turaga D., Blair A.P., Alexanian M., Bruneau B.G. (2021). Co-Emergence of Cardiac and Gut Tissues Promotes Cardiomyocyte Maturation within Human IPSC-Derived Organoids. Cell Stem Cell.

[138] Siller R., Greenhough S., Naumovska E., Sullivan G.J. (2015). Small-Molecule-Driven Hepatocyte Differentiation of Human Pluripotent Stem Cells. Stem Cell Rep..

[139] Lancaster M.A., Huch M. (2019). Disease Modelling in Human Organoids. Dis. Model. Mech..

[140] Kim J., Koo B.K., Knoblich J.A. (2020). Human Organoids: Model Systems for Human Biology and Medicine. Nat. Rev. Mol. Cell Biol..

[141] Sachs N., Clevers H. (2014). Organoid Cultures for the Analysis of Cancer Phenotypes. Curr. Opin. Genet. Dev..

[142] Ren X., Chen W., Yang Q., Li X., Xu L. (2022). Patient-derived cancer organoids for drug screening: Basic technology and clinical application. J. Gastroenterol. Hepatol..

[143] Drost J., Clevers H. (2018). Organoids in Cancer Research. Nat. Rev. Cancer.

[144] National Cancer Institute (NCI) Human Cancer Models Initiative. https://ocg.cancer.gov/programs/hcmi.

[145] Finkbeiner S.R., Zeng X.L., Utama B., Atmar R.L., Shroyer N.F., Estesa M.K. (2012). Stem Cell-Derived Human Intestinal Organoids as an Infection Model for Rotaviruses. mBio.

[146] Ettayebi K., Crawford S.E., Murakami K., Broughman J.R., Karandikar U., Tenge V.R., Neill F.H., Blutt S.E., Zeng X.L., Qu L. (2016). Replication of Human Noroviruses in Stem Cell-Derived Human Enteroids. Science.

[147] Dekkers J.F., Wiegerinck C.L., De Jonge H.R., Bronsveld I., Janssens H.M., De Winter-De Groot K.M., Brandsma A.M., De Jong N.W.M., Bijvelds M.J.C., Scholte B.J. (2013). A Functional CFTR Assay Using Primary Cystic Fibrosis Intestinal Organoids. Nat. Med..

[148] Vlachogiannis G., Hedayat S., Vatsiou A., Jamin Y., Fernández-Mateos J., Khan K., Lampis A., Eason K., Huntingford I., Burke R. (2018). Patient-Derived Organoids Model Treatment Response of Metastatic Gastrointestinal Cancers. Science.

[149] Tiriac H., Belleau P., Engle D.D., Plenker D., Deschênes A., Somerville T.D.D., Froeling F.E.M., Burkhart R.A., Denroche R.E., Jang G.H. (2018). Organoid Profiling Identifies Common Responders to Chemotherapy in Pancreatic Cancer. Cancer Discov..

[150] Hubert C.G., Rivera M., Spangler L.C., Wu Q., Mack S.C., Prager B.C., Couce M., McLendon R.E., Sloan A.E., Rich J.N. (2016). A Three-Dimensional Organoid Culture System Derived from Human Glioblastomas Recapitulates the Hypoxic Gradients and Cancer Stem Cell Heterogeneity of Tumors Found In Vivo. Cancer Res..

[151] Blomme E.A.G., Will Y. (2016). Toxicology Strategies for Drug Discovery: Present and Future. Chem. Res. Toxicol..

[152] Xu H., Jiao Y., Qin S., Zhao W., Chu Q., Wu K. (2018). Organoid Technology in Disease Modelling, Drug Development, Personalized Treatment and Regeneration Medicine. Exp. Hematol. Oncol..

[153] Dijkstra K.K., Cattaneo C.M., Weeber F., Chalabi M., van de Haar J., Fanchi L.F., Slagter M., van der Velden D.L., Kaing S., Kelderman S. (2018). Generation of Tumor-Reactive T Cells by Co-Culture of Peripheral Blood Lymphocytes and Tumor Organoids. Cell.

[154] Diao J., Liu J., Wang S., Chang M., Wang X., Guo B., Yu Q., Yan F., Su Y., Wang Y. (2019). Sweat Gland Organoids Contribute to Cutaneous Wound Healing and Sweat Gland Regeneration. Cell Death Dis..

[155] Sağraç D., Şişli H.B., Şenkal S., Hayal T.B., Şahin F., Doğan A. (2021). Organoids in Tissue Transplantation. Adv. Exp. Med. Biol..

[156] Zhou G., Lieshout R., van Tienderen G.S., de Ruiter V., van Royen M.E., Boor P.P.C., Magré L., Desai J., Köten K., Kan Y.Y. (2022). Modelling Immune Cytotoxicity for Cholangiocarcinoma with Tumour-Derived Organoids and Effector T Cells. Br. J. Cancer.

[157] Singh R.K., Mallela R.K., Cornuet P., Nasonkin I.O. (2015). Derivation of Retinal Cells and Retinal Organoids from Pluripotent Stem Cells for CRISPR-Cas9 Engineering and Retinal Repair. Investig. Ophthalmol. Vis. Sci..

[158] Hoffman B.L., Schorge J.O., Bradshaw K.D., Halvorson L.M., Schaffer J.I., Corton M.M. (2016). Williams Gynecology, 3e.

[159] Kwong J., Franky L.C., Wong K.K., Birrer M.J., Archibald K.M., Balkwill F.R., Berkowitz R.S., Mok S.C. (2009). Inflammatory Cytokine Tumor Necrosis Factor α Confers Precancerous Phenotype in an Organoid Model of Normal Human Ovarian Surface Epithelial Cells. Neoplasia.

[160] Li X., Zheng M., Xu B., Li D., Shen Y., Nie Y., Ma L., Wu J. (2021). Generation of Offspring-Producing 3D Ovarian Organoids Derived from Female Germline Stem Cells and Their Application in Toxicological Detection. Biomaterials.

[161] Wang J., Du H., Ma L., Feng M., Li L., Zhao X., Dai Y. (2023). MitoQ Protects Ovarian Organoids against Oxidative Stress during Oogenesis and Folliculogenesis In Vitro. Int. J. Mol. Sci..

[162] Zhang S., Dolgalev I., Zhang T., Ran H., Levine D.A., Neel B.G. (2019). Both Fallopian Tube and Ovarian Surface Epithelium Are Cells-of-Origin for High-Grade Serous Ovarian Carcinoma. Nat. Commun..

[163] Hill S.J., Decker B., Roberts E.A., Horowitz N.S., Muto M.G., Worley M.J., Feltmate C.M., Nucci M.R., Swisher E.M., Nguyen H. (2018). Prediction of DNA Repair Inhibitor Response in Short Term Patient-Derived Ovarian Cancer Organoids. Cancer Discov..

[164] Hoffmann K., Berger H., Kulbe H., Thillainadarasan S., Mollenkopf H., Zemojtel T., Taube E., Darb-Esfahani S., Mangler M., Sehouli J. (2020). Stable Expansion of High-Grade Serous Ovarian Cancer Organoids Requires a Low-Wnt Environment. EMBO J..

[165] Maenhoudt N., Defraye C., Boretto M., Jan Z., Heremans R., Boeckx B., Hermans F., Arijs I., Cox B., Van Nieuwenhuysen E. (2020). Developing Organoids from Ovarian Cancer as Experimental and Preclinical Models. Stem Cell Rep..

[166] Psilopatis I., Sykaras A.G., Mandrakis G., Vrettou K., Theocharis S. (2022). Patient-Derived Organoids: The Beginning of a New Era in Ovarian Cancer Disease Modeling and Drug Sensitivity Testing. Biomedicines.

[167] Spagnol G., Sensi F., De Tommasi O., Marchetti M., Bonaldo G., Xhindoli L., Noventa M., Agostini M., Tozzi R., Saccardi C. (2023). Patient Derived Organoids (PDOs), Extracellular Matrix (ECM), Tumor Microenvironment (TME) and Drug Screening: State of the Art and Clinical Implications of Ovarian Cancer Organoids in the Era of Precision Medicine. Cancers.

[168] Phan N., Hong J.J., Tofig B., Mapua M., Elashoff D., Moatamed N.A., Huang J., Memarzadeh S., Damoiseaux R., Soragni A. (2019). A Simple High-Throughput Approach Identifies Actionable Drug Sensitivities in Patient-Derived Tumor Organoids. Commun. Biol..

[169] Chen H., Gotimer K., De Souza C., Tepper C.G., Karnezis A.N., Leiserowitz G.S., Chien J., Smith L.H. (2020). Short-Term Organoid Culture for Drug Sensitivity Testing of High-Grade Serous Carcinoma. Gynecol. Oncol..

[170] Lõhmussaar K., Kopper O., Korving J., Begthel H., Vreuls C.P.H., van Es J.H., Clevers H. (2020). Assessing the Origin of High-Grade Serous Ovarian Cancer Using CRISPR-Modification of Mouse Organoids. Nat. Commun..

[171] Wan C., Keany M.P., Dong H., Al-Alem L.F., Pandya U.M., Lazo S., Boehnke K., Lynch K.N., Xu R., Zarrella D.T. (2021). Enhanced Efficacy of Simultaneous PD-1 and PD-L1 Immune Checkpoint Blockade in High-Grade Serous Ovarian Cancer. Cancer Res..

[172] Perets R., Wyant G.A., Muto K.W., Bijron J.G., Poole B.B., Chin K.T., Chen J.Y.H., Ohman A.W., Stepule C.D., Kwak S. (2013). Transformation of the Fallopian Tube Secretory Epithelium Leads to High-Grade Serous Ovarian Cancer in Brca;Tp53;Pten Models. Cancer Cell.

[173] Kessler M., Hoffmann K., Brinkmann V., Thieck O., Jackisch S., Toelle B., Berger H., Mollenkopf H.J., Mangler M., Sehouli J. (2015). The Notch and Wnt Pathways Regulate Stemness and Differentiation in Human Fallopian Tube Organoids. Nat. Commun..

[174] Rose I.M., Bidarimath M., Webster A., Godwin A.K., Flesken-Nikitin A., Nikitin A.Y. (2020). WNT and Inflammatory Signaling Distinguish Human Fallopian Tube Epithelial Cell Populations. Sci. Rep..

[175] Lin Y.X., Wei Y.Z., Jiang M.Z., Tang X., Huang F., Yang X.Z. (2021). Organoid Culture of Mouse Fallopian Tube Epithelial Stem Cells with a Thermo-Reversible Gelation Polymer. Tissue Cell.

[176] Zhang S., Iyer S., Ran H., Dolgalev I., Gu S., Wei W., Foster C.J.R., Loomis C.A., Olvera N., Dao F. (2021). Genetically Defined, Syngeneic Organoid Platform for Developing Combination Therapies for Ovarian Cancer. Cancer Discov..

[177] Yu B., Mccartney S., Strenk S., Valint D.J., Haggerty C., Fredricks D. (2023). Vaginal Bacteria Elicit Acute Inflammatory Response in Fallopian Tube Organoids: A Model for Pelvic Inflammatory Disease. Res. Sq..

[178] Chang Y.H., Chu T.Y., Ding D.C. (2020). Human Fallopian Tube Epithelial Cells Exhibit Stemness Features, Self-Renewal Capacity, and Wnt-Related Organoid Formation. J. Biomed. Sci..

[179] Lancaster M.A., Knoblich J.A. (2014). Organogenesis in a Dish: Modeling Development and Disease Using Organoid Technologies. Science.

[180] Haider S., Gamperl M., Burkard T.R., Kunihs V., Kaindl U., Junttila S., Fiala C., Schmidt K., Mendjan S., Knöfler M. (2019). Estrogen Signaling Drives Ciliogenesis in Human Endometrial Organoids. Endocrinology.

[181] Jamaluddin M.B.F.F.B., Ghosh A., Ingle A., Mohammed R., Ali A., Bahrami M., Kaiko G., Gibb Z., Filipe E.C., Cox T.R. (2022). Bovine and Human Endometrium-Derived Hydrogels Support Organoid Culture from Healthy and Cancerous Tissues. Proc. Natl. Acad. Sci. USA.

[182] Filby C.E., Wyatt K.A., Mortlock S., Cousins F.L., McKinnon B., Tyson K.E., Montgomery G.W., Gargett C.E. (2021). Comparison of Organoids from Menstrual Fluid and Hormone-Treated Endometrium: Novel Tools for Gynecological Research. J. Pers. Med..

[183] Murphy A.R., Campo H., Kim J.J. (2022). Strategies for Modelling Endometrial Diseases. Nat. Rev. Endocrinol..

[184] Juárez-barber E., Francés-herrero E., Corachán A., Vidal C., Giles J., Alamá P., Faus A., Pellicer A., Cervelló I., Ferrero H. (2022). Establishment of Adenomyosis Organoids as a Preclinical Model to Study Infertility. J. Pers. Med..

[185] Esfandiari F., Favaedi R., Heidari-Khoei H., Chitsazian F., Yari S., Piryaei A., Ghafari F., Baharvand H., Shahhoseini M. (2021). Insight into Epigenetics of Human Endometriosis Organoids: DNA Methylation Analysis of HOX Genes and Their Cofactors. Fertil. Steril..

[186] Girda E., Huang E.C., Leiserowitz G.S., Smith L.H. (2017). The Use of Endometrial Cancer Patient–Derived Organoid Culture for Drug Sensitivity Testing Is Feasible. Int. J. Gynecol. Cancer.

[187] Katcher A., Yueh B., Ozler K., Nizam A., Kredentser A., Chung C., Frimer M., Goldberg G.L., Beyaz S. (2023). Establishing Patient-Derived Organoids from Human Endometrial Cancer and Normal Endometrium. Front. Endocrinol..

[188] Jamaluddin M.F.B., Ko Y.A., Ghosh A., Syed S.M., Ius Y., O’Sullivan R., Netherton J.K., Baker M.A., Nahar P., Jaaback K. (2022). Proteomic and Functional Characterization of Intra-Tumor Heterogeneity in Human Endometrial Cancer. Cell Rep. Med..

[189] Tamura H., Higa A., Hoshi H., Hiyama G., Takahashi N., Ryufuku M., Morisawa G., Yanagisawa Y., Ito E., Imai J.I. (2018). Evaluation of Anticancer Agents Using Patient-Derived Tumor Organoids Characteristically Similar to Source Tissues. Oncol. Rep..

[190] Rawlings T.M., Makwana K., Tryfonos M., Lucas E.S. (2021). Organoids to Model the Endometrium: Implantation and Beyond. Reprod. Fertil..

[191] Murphy A.R., Wiwatpanit T., Lu Z., Davaadelger B., Kim J.J. (2019). Generation of Multicellular Human Primary Endometrial Organoids. J. Vis. Exp..

[192] Jones R.E., Lopez K.H. (2014). The Female Reproductive System. Human Reproductive Biology.

[193] Chumduri C., Turco M.Y. (2021). Organoids of the Female Reproductive Tract. J. Mol. Med..

[194] Chumduri C., Gurumurthy R.K., Berger H., Dietrich O., Kumar N., Koster S., Brinkmann V., Hoffmann K., Drabkina M., Arampatzi P. (2021). Opposing Wnt Signals Regulate Cervical Squamocolumnar Homeostasis and Emergence of Metaplasia. Nat. Cell Biol..

[195] Lõhmussaar K., Oka R., Espejo Valle-Inclan J., Smits M.H.H., Wardak H., Korving J., Begthel H., Proost N., van de Ven M., Kranenburg O.W. (2021). Patient-Derived Organoids Model Cervical Tissue Dynamics and Viral Oncogenesis in Cervical Cancer. Cell Stem Cell.

[196] Ramirez-Gonzalez J.A., Vaamonde-Lemos R., Cunha-Filho J.S., Varghese A.C., Swanson R.J., Vaamonde D., du Plessis S.S., Agarwal A. (2016). Overview of the Female Reproductive System. Exercise and Human Reproduction.

[197] Ali A., Syed S.M., Jamaluddin M.F.B., Colino-Sanguino Y., Gallego-Ortega D., Tanwar P.S. (2020). Cell Lineage Tracing Identifies Hormone-Regulated and Wnt-Responsive Vaginal Epithelial Stem Cells. Cell Rep..

[198] Xiang N., Ni Z. (2023). Microfluidics for Biomedical Applications. Biosensors.

[199] Sackmann E.K., Fulton A.L., Beebe D.J. (2014). The Present and Future Role of Microfluidics in Biomedical Research. Nature.

[200] Hu Q., Luni C., Elvassore N. (2018). Microfluidics for Secretome Analysis under Enhanced Endogenous Signaling. Biochem. Biophys. Res. Commun..

[201] Xiao S., Coppeta J.R., Rogers H.B., Isenberg B.C., Zhu J., Olalekan S.A., McKinnon K.E., Dokic D., Rashedi A.S., Haisenleder D.J. (2017). A Microfluidic Culture Model of the Human Reproductive Tract and 28-Day Menstrual Cycle. Nat. Commun..

[202] Young R.E., Huh D.D. (2021). Organ-on-a-Chip Technology for the Study of the Female Reproductive System. Adv. Drug Deliv. Rev..

